# Use of short interfering RNA delivered by cationic liposomes to enable efficient down-regulation of *PTPN22* gene in human T lymphocytes

**DOI:** 10.1371/journal.pone.0175784

**Published:** 2017-04-24

**Authors:** Valentina Perri, Marsha Pellegrino, Francesca Ceccacci, Anita Scipioni, Stefania Petrini, Elena Gianchecchi, Anna Lo Russo, Serena De Santis, Giovanna Mancini, Alessandra Fierabracci

**Affiliations:** 1 Type 1 Diabetes Centre, Infectivology and Clinical Trials Area, Children’s Hospital Bambino Gesù, Rome, Italy; 2 CNR Chemical Methodologies Institute-Section Mechanisms of reaction (CNR-IMC-SMR) c/o Sapienza University, Rome, Italy; 3 Department of Chemistry, Sapienza University, Rome, Italy; 4 Confocal Microscopy Core Facility, Research Laboratories, Children’s Hospital Bambino Gesù, Rome, Italy; 5 CNR Chemical Methodologies Institute (CNR-IMC), Rome, Italy; Charles University, CZECH REPUBLIC

## Abstract

Type 1 diabetes and thyroid disease are T cell-dependent autoimmune endocrinopathies. The standard substitutive administration of the deficient hormones does not halt the autoimmune process; therefore, development of immunotherapies aiming to preserve the residual hormonal cells, is of crucial importance. *PTPN22* C1858T mutation encoding for the R620W lymphoid tyrosine phosphatase variant, plays a potential pathophysiological role in autoimmunity. The *PTPN22* encoded protein Lyp is a negative regulator of T cell antigen receptor signaling; R620W variant, leading to a gain of function with paradoxical reduced T cell activation, may represent a valid therapeutic target. We aimed to develop novel wild type *PTPN22* short interfering RNA duplexes (siRNA) and optimize their delivery into Jurkat T cells and PBMC by using liposomal carriers. Conformational stability, size and polydispersion of siRNA in lipoplexes was measured by CD spectroscopy and DLS. Lipoplexes internalization and toxicity evaluation was assessed by confocal microscopy and flow cytometry analysis. Their effect on Lyp expression was evaluated by means of Western Blot and confocal microscopy. Functional assays through engagement of TCR signaling were established to evaluate biological consequences of down-modulation. Both Jurkat T cells and PBMC were efficiently transfected by stable custom lipoplexes. Jurkat T cell morphology and proliferation was not affected. Lipoplexes incorporation was visualized in CD3+ but also in CD3- peripheral blood immunotypes without signs of toxicity, damage or apoptosis. Efficacy in affecting Lyp protein expression was demonstrated in both transfected Jurkat T cells and PBMC. Moreover, impairment of Lyp inhibitory activity was revealed by increase of IL-2 secretion in culture supernatants of PBMC following anti-CD3/CD28 T cell receptor-driven stimulation. The results of our study open the pathway to future trials for the treatment of autoimmune diseases based on the selective inhibition of variant *PTPN22* allele using lipoplexes of siRNA antisense oligomers.

## Introduction

Autoimmune thyroid diseases (ATD) [1,2] and insulin-dependent diabetes mellitus (Type 1 diabetes, T1DM) [3] are due to target cell destruction by autoreactive T lymphocytes [4]. This disease combination is named autoimmune polyglandular syndrome Type 3 variant (APS3v) [5]. There is an increased incidence of autoimmunity and T1DM worldwide especially in children under 5 years of age, likely associated with ATD [6]. The substitutive administration of the deficient hormones i.e. insulin [7] and levo-thyroxine (L-T4) [8] is the standard treatment that, however, does not halt the autoimmune process and does not rescue the residual hormone producing cells. Identification of innovative therapeutic interventions, especially aimed to preserve the residual hormone producing cells, is of crucial importance in the expectation of quality of life in pediatric patients [9]. Family and population studies have shown that APS3v has a strong genetic background [10]. Whole genome and candidate gene approaches have identified several gene variations that are present in both ATD and T1DM ([11], reviewed in [12]). Recently, particular interest was generated by the potential pathophysiological role played in several autoimmune conditions including T1DM and APS3v [13] by the *PTPN22* (protein tyrosine phosphatase N22 gene) C1858T mutation, which changes amino acid residue 620 from Arg (R) to Trp (W) (R620W) in the lymphoid tyrosine phosphatase Lyp protein. This is a negative regulator of T cell antigen receptor (TCR) signaling, acting in concert with C-terminal Src kinase (CSK). R620W variant leads to a gain of function mutation with paradoxical reduced T cell activation. Peripheral T lymphocytes of T1DM patients are indeed hyporesponsive to *in vitro* stimulation with monoclonal antibodies (mAbs) to CD3 (anti-CD3) [14]. Subtle TCR signaling defects induced by Lyp variant could have implications at the level of thymocyte tolerisation and escape of autoreactive T lymphocytes [15], through positive selection of otherwise negatively selected autoimmune T cells. The variant has effect on both innate and adaptive immune responses [16]. Recently we observed altered B cell homeostasis and Toll-Like receptor (TLR) 9-driven response in T1DM carriers of the *PTPN22* C1858T allelic variant (rev. in [17,18]). In the light of this, LypR620W may be a valid drug target for the treatment of T1DM and APS3v patients.

As regards one of the approaches to immunotherapy, gene silencing can be achieved by using antisense oligonucleotides (ASO), ribozymes, DNAzymes or RNA interference (RNAi) ([19] and reviewed in [20]). On a theoretical basis, RNAi could represent a safer and effective new class of therapeutics with potential translational application in treating disease, taking advantage of the body’s own natural processes to silence genes by eliminating specific gene-products or proteins in the cell [21–24]. siRNAs, constituted by two antisense strands intended to recognize a target RNA, have proven to be a more robust technology to achieve effective silencing in cultured cells than ASOs having one strand [22]. Systemic injection of ASO was already shown to be a feasible approach for treating disease-related genes [25]. However, with specific reference to immunotherapies, the utility of antisense technology already presented limitations not only for molecules high extent of degradation but also for their inefficient transport across the plasma membranes of immunocytes, in particular T and B lymphocytes [26]. For this reason, a crucial goal in the development of a feasible immunotherapy is the realization of delivery agents capable of transporting safely and effectively the siRNAs [22]. Both viral and non-viral carriers for gene therapy have been developed and used in clinical trials [24]. However, among the developed systems, viral delivery agents present several limitations, i.e. carcinogenesis, immunogenicity, and difficulty of production. Non-viral agents seem to address some of these limitations since they are easier to prepare and less toxic with respect to viral ones. Moreover, they are easy to scale up and load with a larger amount of genetic payload; they also show physico-chemical stability, high reproducibility and efficiency *in vitro*, although a lower transfection *in vivo* efficiency is reported. The most studied non-viral delivery agents are liposomes, polymeric and lipid nanoparticles, micelles, dendrimers [27] and carbon nanotubes [28].

Engineered nanocarriers were developed to target T and B lymphocytes without toxic side effects. In particular ASO carried by PEGylated carbon nanotubes (PNT) [29] enable efficient knockdown of *PTPN22* gene in T lymphocytes [20]. Although PNT were shown of limited toxicity in mice [30] these carriers lack approval by the Food and Drug Administration (FDA, Rockville, MD) for delivery in humans.

In the light of the foregoing, the goal of the present study was to develop novel *PTPN22* short interfering RNA duplexes (siRNA) and optimize their T cell delivery by using liposomal carriers. Liposomes have a long history as drug carriers because of their easy preparation, minimal toxicity and biodegradability profiles [31,32]. The first liposome formulation approved by FDA in 1995 was Doxil and used to treat ovarian cancer, AIDS (acquired immunodeficiency syndrome)-related Kaposi’s sarcoma and multiple myeloma [33]. Liposomal formulations have also been approved for anticancer treatment in pediatric patients [34] or in systemic fungal infections [35]. Safety and efficacy of siRNA-liposomal formulations have been already demonstrated in human trials, therefore tolerability is ascertained and toxicity is generally excluded [21–23,36–39]. Liposomes are very versatile delivery systems as their physico-chemical properties (size, charge, membrane fluidity) can be easily tuned by choosing the suitable composition (nature of components and their ratio). Such versatility allows, in turn, the modulation of the properties of the liposome/siRNA complex [27].

In this work, we used cationic liposomes composed of the natural lipid dimyristoyl-*sn*-glycero-phosphatidylcholine (DMPC) and a synthetic cationic gemini surfactant (2*S*,3*S*-2,3-dimethoxy-1,4-bis(N-hexadecyl-N,N-dimethylammonium)-butane dibromide (1) or 2*R*,3*S*-2,3-dimethoxy-1,4-bis(N-hexadecyl-N,N-dimethylammonium)-butane dibromide (2) [40–43]. The two gemini surfactants differ for the stereochemistry of the spacer. The transfection efficiency of both was tested since it was already reported that their stereochemistry affects delivery and transfection efficiency of liposomes in which they are embedded [32,41]. In unravelling possible new pathways to future trials for the treatment of autoimmune diseases in humans, our approach aimed to allow T cell specific down-regulation by using antisense strand complementary to the mRNA of *PTPN22*.

## Materials and methods

### Preparation and characterization of liposome formulations

Gemini surfactants 2S,3S-2,3-dimethoxy-1,4-bis(N-hexadecyl-N,N-dimethylammonium)-butane dibromide (1) and 2R,3S-2,3-dimethoxy-1,4-bis(N-hexadecyl-N,N-dimethylammonium)-butane dibromide (2) were prepared as previously described [40,44] (S1 Fig).

The aqueous dispersions of DMPC (purity>99%, Avanti Polar Lipids Inc. (Alabaster, AL) and gemini were prepared according to a previously described procedure [45]. Briefly, a film of lipids (total 6.0 μmol) was prepared on the inside wall of a round-bottom flask by evaporation of a CHCl_3_ solution containing the proper amounts of DMPC and gemini to obtain the 50/50 molar percentage mixture. The obtained films were dried overnight (O/N) under high vacuum, and 3.0 ml of buffer solution (5mM HEPES, 0.1 mM EDTA, pH 7.4 (Sigma-Aldrich, Chemical Company (Co.), St Louis, MO) were added in order to obtain 1.0 mM lipid dispersions. The solutions were vortex-mixed and then freeze-thawed six times from liquid nitrogen to 313 K. Dispersions were then extruded (10 times) through a 100 nm polycarbonate membrane (Whatman Nuclepore, Toronto, ON, Canada). The extrusions were carried out at 40°C, well above the transition temperature of DMPC (24.2°C), using a 10 ml extruder (Lipex Biomembranes, Vancouver, Canada).

Proper volumes of a siRNA solution (0.1 mM in buffer) were added to a diluted liposome solution to have the final concentrations: [siRNA] = 1.3 μM, [DMPC] = 50 μM, [gemini] = 50 μM, corresponding to a charge ratio +/- = 2.

Liposome formulations for confocal microscopy were prepared following the same procedure described above, adding phospholipids functionalized with a fluorescent probe to the lipid solution, during the film preparation step. The probe 1,2-dimyristoyl-*sn*-glycero-3-phosphoethanolamine-N-(lissamine rhodamine B sulfonyl) (ammonium salt, Avanti Polar Lipids) was used in a 0.1 mol percentage with respect to DMPC.

Liposomal suspensions were analysed by circular dichroism spectroscopy (CD) and dynamic light scattering (DLS) at different times after the preparation (9, 24, 48, 72 hours).

#### Circular dichroism spectroscopy

CD spectra were recorded on a Jasco spectropolarimeter J-715 equipped with a Peltier device for the temperature control, using a quartz cuvette of 0.5 cm path length. Measurements were run in the 330–220 nm spectral range at 25°C. CD spectra are the average of 16 scans obtained with an instrument scanning speed of 100 nm/min, response time of 1 second (s) and resolution of 1 nm.

#### Dynamic light scattering

DLS measurements were obtained with a Brookhaven Instruments Corp.BI-200SM goniometer equipped with a BI-9000AT digital correlator using a solid-state laser (125 mW, λ = 532 nm). If not otherwise stated, measurements of scattered light were made at a scattering angle θ of 90°. The temperature was controlled with an accuracy of 0.1°C. Experiment duration was in the range of 5–20 minutes, and each experiment was repeated two or more times. CONTIN algorithm was used to fit the data. Measurements were carried out at 25°C.

### Cell cultures

Jurkat T cells (American Type Culture Collection (ATCC^®^ TIB-152^™^ USA, S2 Fig) were cultured in T75 flasks (Falcon Labware Becton Dickinson (BD) Biosciences, Oxnard, CA) in complete RPMI 1640 medium (GIBCO/BRL, Invitrogen, Gaithersburg, CA) supplemented with 10% FBS (Hyclone, South Logan, UT), L-glutamine (2 mM, EuroClone Milan, Italy) and 1% penicillin/streptomycin (pen/strep) (Euroclone) and cultured at 37°C in a humidified atmosphere containing 5% CO_2_.

Peripheral blood mononuclear cells (PBMC) were separated by Ficoll-Hypaque (Histopaque, Sigma-Aldrich) from sodium heparinized venous blood samples (5–10 ml) of 4 healthy blood volunteer donors (HD) recruited in our laboratory according to standard protocols [18]. The study was approved by the local Institutional Review Board (IRB) of Bambino Gesu`Children’s Hospital, regulating the use of human samples for experimental studies (Protocol number 669 LB, 17^th^ July 2015). Written informed consent was obtained from participants. Subjects DNA harbored wild type *PTPN22* gene alleles when tested using a restriction fragment length polymorphism-polymerase chain reaction (PCR) method [reviewed in 17].

### *PTPN22* gene silencing of Jurkat T cells

#### siRNA design and transfection with commercial transfection system

siRNA sequences were specifically and originally designed for the target *PTPN22* wild-type gene. These have been created using a siRNA design algorithm licensed from Rosetta Inpharmatics (Sigma Aldrich Chemical Co., http://www.sigmaaldrich.com/life-science/functional-genomics-and-rnai/siRNA/learning-center/mission-sup-reg0/siRNA-design-choosing.html). From a list of siRNA sense/antisense (s/a) duplexes differing in mRNA target affinity (S1 Table), generated without any backbone modification) we subsequently validated a set by testing their ability of silencing in Jurkat T cells by using a commercial transfection system (Santa Cruz Biotechnology, Dallas, TX) and observing the resulting decrease in target protein levels on a Western Blot (WB) assay. From the data obtained, we chose the specific sequence, which happened to be the one with the higher affinity for the target, for our subsequent experiments, namely *PTPN22* siRNA s/a duplex (SNP_C sense 5’-GUACGGACACCUGAAUCAUdTdT-3’; SNP_C antisense 5’ AUGAUUCAGGUGUCCGUACdTdT-3’, Sigma Chemical Co.) (siRNA1).

For the transfection procedure on day 1, Jurkat T cells were harvested from culture flasks, washed in phosphate buffered saline (PBS, Lonza, Verviers, Belgium) and plated at 1.5 x 10^6^ per well in 24-well plates (Falcon, Corning Incorporated, Corning NY) in 1 ml of RPMI 1640 medium (GIBCO/BRL) supplemented only with L-glutamine (2 mM) and incubated for 24 hours at 37°C in a humidified atmosphere containing 5% CO_2_.

On day 2, Jurkat cells were collected, washed again in a solution containing siRNA transfection medium (Santa Cruz Biotechnology) and plated in a volume of 250 μl in 48-well plates (Falcon) with different quantities of *PTPN22* siRNA s/a in transfection medium (1:50; 2:50; 3:50; 4:50 corresponding to 20, 40, 60, 80 pmols of siRNA) and incubated for 7 hours at 37°C in humidified atmosphere containing 5% CO_2_. For control of internalization efficiency cells were incubated with the fluorescent (FITC-conjugated) siRNA provided by the manufacturer. The 7-hour incubation was stopped by adding complete RPMI supplemented with 20% FBS and 2% pen/strep. The cells were cultured for additional 18–24 hours. The control group was treated with the fluorescent siRNA, it was subsequently harvested and analyzed by flow-cytometry on FACSCanto II instrument (Becton and Dickinson, Sunnyvale, CA) and PC FACSDiva software (BD Biosciences, San Jose, CA) to assess siRNA internalization efficacy.

On day 3, the cells were harvested, washed in PBS and seeded in 1 ml of complete RPMI medium in 24-well culture plates. The timing for the ultimate cell harvest started from this moment. On day 4, after 24 hours, and day 5, after 48 hours, the experimental sets of cells were harvested, washed with PBS and centrifuged at 1500 rpm for 5 minutes. Cell pellets were frozen at -80°C.

#### Custom liposome transfection protocol for Jurkat T cells

On day 1, Jurkat cells were collected from cultures, washed in PBS and additionally cultured in T75 flasks in RPMI 1640 medium supplemented with 10% FBS and L-glutamine (2 mM) and incubated for 24 hours at 37°C in a humidified atmosphere containing 5% CO_2_.

On day 2, cells were harvested from flasks, washed in PBS by centrifugation at 1200 rpm for 5 minutes and plated with the same FBS free medium at 1.5 x 10^6^ per well in 48-well plates (Falcon) in a final volume of 250 μl. this last one containing the siRNA duplex against the *PTPN22* gene (*vide supra*) complexed with liposomes (lipoplexes Lipo/siRNA). In a first set of experiments, lipoplexes were used at 20, 40, 60, 80 and 100 pmols doses of siRNA. Cells were incubated with the complexes for 60, 90 minutes or O/N at 37°C in humidified atmosphere containing 5% CO_2_. Appropriate controls were set up by growing cells with RPMI medium alone, RPMI medium supplemented with empty liposomes (50 pmols), or with the siRNA not complexed with liposomes at the indicated dose. Each experimental condition included at least three independent determinations (triplicates). At the end of the incubation period cells were harvested, washed in PBS by centrifugation at 1200 rpm for 5 minutes and plated in complete RPMI medium in 24-well plates at a final volume of 1 ml.

On day 4, after 48 hours, and on day 5, after 72 hours from the beginning of each transfection, cells were harvested and washed in PBS by centrifugation at 1500 rpm for 5 minutes. Pellets were collected and frozen at -80°C for subsequent analysis.

### *PTPN22* gene silencing in human PBMC

#### Custom liposome transfection protocol

Preliminary experiments were performed to test the efficacy of the same high affinity Lipo/siRNA duplex on liquid-nitrogen frozen PBMC of HD samples. PBMC were washed with complete RPMI, plated at 1.5 x 10^6^ cells per well in a 48-well plate at a final volume of 250 μl/well and transfected O/N with lipoplexes containing 60, 80 and 100 pmols of siRNA. Control cells were administered with either empty liposomes or not complexed siRNA.

### Western blot analysis

Total protein extraction was performed by resuspending cell pellets in ice-cold Ripa lysis buffer [50 mM Tris pH 7.5, 150 mM NaCl, 1% Triton X-100, 1 mM EGTA, 1% sodium deoxycholate and phosphatases 1% cocktail protease inhibitors (Sigma-Aldrich Co.), 0.5 mM sodium orthovanadate]. Proteins were separated on 10% SDS-PAGE and transferred onto a nitrocellulose membrane (Amersham Hybond ECL, GE Healthcare, Germany). After blocking with 5% nonfat dry milk (NFDM) (Cell Signaling Technology, MA) in TBS (Tris-buffered saline)-T (Tween 20) [0.1% T (pH 7.5)] membranes were incubated with the following primary antibodies appropriately diluted in 3% NFDM TBS-T: monoclonal mouse IgG_2B_ anti-human Lyp (Clone #340113, 1:500; R&D Systems Minneapolis, MN) and rabbit anti-human β-actin (1:2000; Santa Cruz Biotechnology). After washing 3x10 minutes in TBS-T, membranes were incubated with secondary antibodies directed to mouse IgG (1:5000; Jackson Immuno Research, UK) or rabbit IgG (1:10000; Santa Cruz Biotechnology) respectively in 3% NFDM TBS-T. Specific bands of 105 KDa for Lyp and 43 KDa for β-actin were detected using the chemiluminescence system (ECL™) Pierce ECL Western Blotting Substrate (Thermo Scientific, Rockford, IL). The relative intensities of the Lyp specific bands were recorded with ChemiDoc apparatus (Bio-Rad Laboratories, Hercules, CA) and quantified by densitometric analysis referring to β-actin protein expression using Image Lab Software version 3.0 (Bio-Rad Laboratories).

### Confocal microscopy analysis

#### Detection of liposome/siRNA complex in Jurkat T cells

Lipo/siRNA complexes (100 pmols of siRNA) were marked with rhodamine (as regards the preparation of marked lipoplexes, see the paragraph ‘Preparation and characterization of liposome formulations’) and were incubated with Jurkat T cells plated in 48 well culture plates (*vide supra*) for 30, 60, 90 minutes and 4 and half hours. At the end of the incubation period cells were harvested, washed in PBS and fixed with 4% paraformaldehyde (Sigma-Aldrich Chemical Co.). Fixed cell suspension was distributed dropwise onto positive charged microscope slides (Super Frost plus, Menzel-Glaser, Germany) and dried at 37°C. After rehydration in PBS cell preparations were stained with wheat germ agglutinin (WGA conjugated to Oregon Green® 488 (1:200) Invitrogen Molecular Probes, Eugene, OR) diluted in PBS supplemented with 1% bovine serum albumin (BSA). Nuclei were counterstained with 1 μg/ml Hoechst 33342 (Invitrogen). Confocal imaging was performed on an Olympus Fluoview FV1000 confocal microscope equipped with FV10-ASW version 2.0 software, Multi Ar (458–488 and 515 nm), 2× He/Ne (543 and 633 nm), and 405-nm diode lasers, using a 60× (1.40 NA oil) objective. Optical single sections were acquired with a scanning mode format of 1024×1024 pixels, sampling speed of 40 ms/pixel (pixel size of 0.2 mm), and Z-reconstructions of serial single optical sections were carried out with an electronic zoom at 2.5. Fluorochromes unmixing was performed by acquisition of automated-sequential collection of multi-channel images, in order to reduce spectral crosstalk between channels.

#### Detection of Lyp protein in transfected Jurkat T cells

After 48 and 72 hours from the beginning of an O/N transfection with Lipo/siRNA complexes (100 pmols of siRNA), Jurkat T cells were harvested, washed in PBS, fixed in 4% paraformaldehyde, and stained with monoclonal mouse IgG_2B_ anti-human Lyp antibody (R&D Systems). Fixed cell suspensions were distributed dropwise onto positive charged microscope slides (Menzel-Glaser). Cell permeabilization was obtained incubating microscope slides with 0.1% PBS-Triton X-100 (Sigma-Aldrich Co.) for 5 minutes. After blocking with 5% normal goat serum (NGS, Sigma-Aldrich Co.) in PBS/BSA for 30 minutes cells were incubated O/N at 4°C with monoclonal mouse anti-human Lyp antibody (Clone #340113, 1:100; R&D Systems). Microscope slides were then washed in PBS and incubated with goat anti-mouse IgG Alexa Fluor^®^ 555 conjugate (1:500; Invitrogen) secondary antibody for 1 hour at room temperature (RT). All antibodies were diluted in PBS/1% BSA. Nuclei were counterstained with Hoechst 33342 and cells membranes were stained with WGA Oregon Green^®^ 488 conjugate as described above.

#### Detection of liposome/siRNA complex in human PBMC

Lipo/siRNA complexes (100pmols of siRNA) marked with rhodamine were incubated with PBMC cultured in 48-well culture plates for 4 and a half hours. Paraformaldehyde-fixed cell suspensions were located on microscope slides as described above. Subsequently, samples were stained with primary purified mouse anti-human CD3 antibody (BD Biosciences 1:30, incubated for 1 hour at RT) followed by secondary antibody goat anti-mouse Cy-5 conjugate (Invitrogen 1:100, incubated for 1 hour at RT). WGA conjugated to Oregon Green® 488 (Invitrogen 1:200,) and 1 **μ**g/ml Hoechst 33342 (Invitrogen) were used to counterstain plasma membrane and nucleic acid respectively. In additional stainings the following antibodies were used: FITC (fluorescein conjugated) mouse anti-human CD56 (BD Bioscience 1:30, incubated for 1 hour at RT); APC (allophycocyanin conjugated) mouse anti-human CD86 (BD Bioscience 1:10, incubated for 1 hour at RT); Alexa Fluor 700 mouse anti-human CD19 (BD Bioscience 1:30, incubated for 1 hour at RT); FITC goat anti-mouse secondary antibody (Invitrogen 1:500, incubated for 1 hour at RT); 1 **μ**g/ml Hoechst 33342 (Invitrogen) was used to counterstain nuclei.

#### Detection of Lyp protein in transfected human PBMC

Lipo/siRNA complexes (100pmols of siRNA) marked with rhodamine were incubated with PBMC cultured in 48-well culture plates for 4 and a half hours. Paraformaldehyde-fixed cell suspensions were located on microscope slides as described above. For comparing Lyp protein expression among CD3^**+**^ and CD3^**-**^ cells the following antibodies were used: monoclonal mouse IgG_**2B**_ anti-human Lyp antibody (Clone #340113, 1:100; R&D Systems, O/N at 4°C), Alexa fluor 488 goat anti-mouse IgG2b secondary antibody (Invitrogen, 1:800 incubated for 1 hour at RT); mouse anti-human CD3 primary antibody (BD Biosciences 1:30, incubated for 1 hour at RT); Alexa Fluor 647 goat anti-mouse IgG1 secondary antibody (Invitrogen, 1:800 incubated for 1 hour at RT). Nuclei were counterstained with 1 μg/ml Hoechst 33342 (Invitrogen) as above described.

### Toxicity evaluation of lipoplexes on Jurkat T cells and PBMC

Toxicity evaluation of lipoplexes was evaluated by monitoring cell morphology, viability, quantity and quality of cell pellets and quantification of protein extract concentration at the end of the experimental procedure. For Jurkat T cells, proliferative assays were also established according to the following protocol. Before transfection, Jurkat T cells were labelled with 5-chloromethylfluorescein diacetate (CMFDA) (CellTracker Green, Invitrogen, Molecular Probes, OR) at a final concentration of 0.1 **μ**g/ml and plated at 5 × 10^5^ cells per well in 96-well plates (Falcon) in FBS-free RPMI 1640 (GIBCO/BRL) supplemented with L-glutamine (2 mM,) at a final volume of 200 **μ**l/well. Otherwise they were treated with DMPC2 (liposome formulation), siRNA 100pmols or DMPC/2/siRNA100pmols (siRNA complexed with liposomes formulation). After an O/N incubation at 37°C in humidified atmosphere containing 5% CO_2_, cells were harvested, washed in PBS by centrifugation at 1200 rpm for 5 minutes and cultured in complete RPMI medium supplemented with 10% FBS, L-glutamine (2 mM) and 1% pen/strep. Cell proliferation was assessed after 48 hours and 72 hours from the beginning of the transfection by fluorescence-activated cell sorting analysis (FACS) using FACSCanto II instrument (BD, Sunnyvale, CA) and FACSDiva software for data analysis (BD Biosciences, San Jose, CA). Ten thousand events per sample were analysed.

For evaluation of cell death Jurkat T cells and HD PBMC were seeded at 1.5 x 10^6^ cells per well in 48-well plates (Falcon) in a final volume of 250 **μ**l of FBS-free RPMI 1640 medium (GIBCO/BRL) supplemented with L-glutamine (2 mM) (EuroClone) and treated with different doses of DMPC/2/siRNA marked with rhodamine (20, 60, 80, 100 pmols of siRNA) for 4 and a half hours. Subsequently the cells were harvested with complete medium, centrifuged 1200 rpm for 5 minutes, washed once in PBS and resuspended in PBS 2% FBS. To detect and quantify dead cells the blue fluorescent cell impermeant dye DAPI (Invitrogen) was added at a final concentration of 0,2 **μ**M, 5 minutes prior to analyzing cells by flow-cytometer BD LSR Fortessa X-20 (BD, Sunnyvale, CA). DAPI specifically enters only dead cells when utilized on a live non-fixed cell sample. Twenty thousand events were acquired and data analyzed by BD FACSDiva software 8.0 (BD, San Jose, CA).

### Functional assays

We implemented a functional assay evaluating IL-2 concentration in supernatants of Jurkat T cells transfected O/N with Lipo/siRNA complexes (with 60 or 100 pmols of siRNA) to verify their effect on T cell activation. Thus after transfection, cells were washed by centrifugation, seeded at 2 x 10^5^ in 96 flat bottomed plates in complete RPMI medium (*vide supra*) and activated with the addition of Dynabeads Human T-activator CD3/CD28 beads (Invitrogen) at a bead-to-cell-ratio of 1:1 to engage TCR signaling. Subsequently they were incubated at 37°C in a humidified atmosphere containing 5% CO_2_ for 48 hours.

In parallel experiments, after 48 hours from the beginning of the transfection, Jurkat T cells were activated with 50 ng/ml phorbol myristate acetate (PMA, Invitrogen) and 1 **μ**g/ml ionomycin (Sigma-Aldrich) [13,46] for 24 and 48 hours.

The same protocol was used to evaluate IL-2 concentration in supernatants of PBMC derived from two different healthy volunteers (HD 1 and 2) transfected O/N with Lipo/siRNA complexes (with 60 pmols of siRNA) and stimulated for 5 days with Dynabeads Human T-activator CD3/CD28 beads (Invitrogen) at a bead-to-cell-ratio of 1:50. This suboptimal stimulus was proven ideal in experiments investigating immunomodulatory activities [47].

At the end of the incubation periods, plates from all datasets were centrifuged at 1200 rpm for 5 minutes and supernatants were collected. The concentration of IL-2 in supernatants was estimated by means of the human IL-2 ELISA development kit (Mabtech, Nacka strand, Sweden) following the manufacturer’s guidelines. Plates were then read at 405 nm by using Benchmark Plus microplate spectrophotometer (Bio-Rad, CA).

### Statistical analysis

Differences of protein levels between lipoplexes treated and control groups were at first tested for normality with the Kolmogorov-Smirnov test and subsequently analyzed (with KS-test: p>0.10) for statistical significance with one-way ANOVA and Tukey’s Multiple Comparison Test. Results of multiple biological determinations were analyzed using GraphPad Prism software version number 5 (San Diego, CA). Differences in siRNA+ cells among CD3+ and CD3- groups, representing the population’s specific transfection efficiency, were statistically evaluated with the same software using the unpaired t test. The analysis was performed by scoring a total of 1546 cells by two independent observers (MP, SP). The number of microscopy fields analyzed was 13. A result with p<0.05 was considered statistically significant. Unpaired t test was used to compare results of Lyp protein expression levels after siRNA1 and siRNA2 transfection using the commercial system and of IL-2 secretion in functional assays.

## Results

### CD investigations into the conformational stability of siRNA in lipoplexes

**Fig 1:**
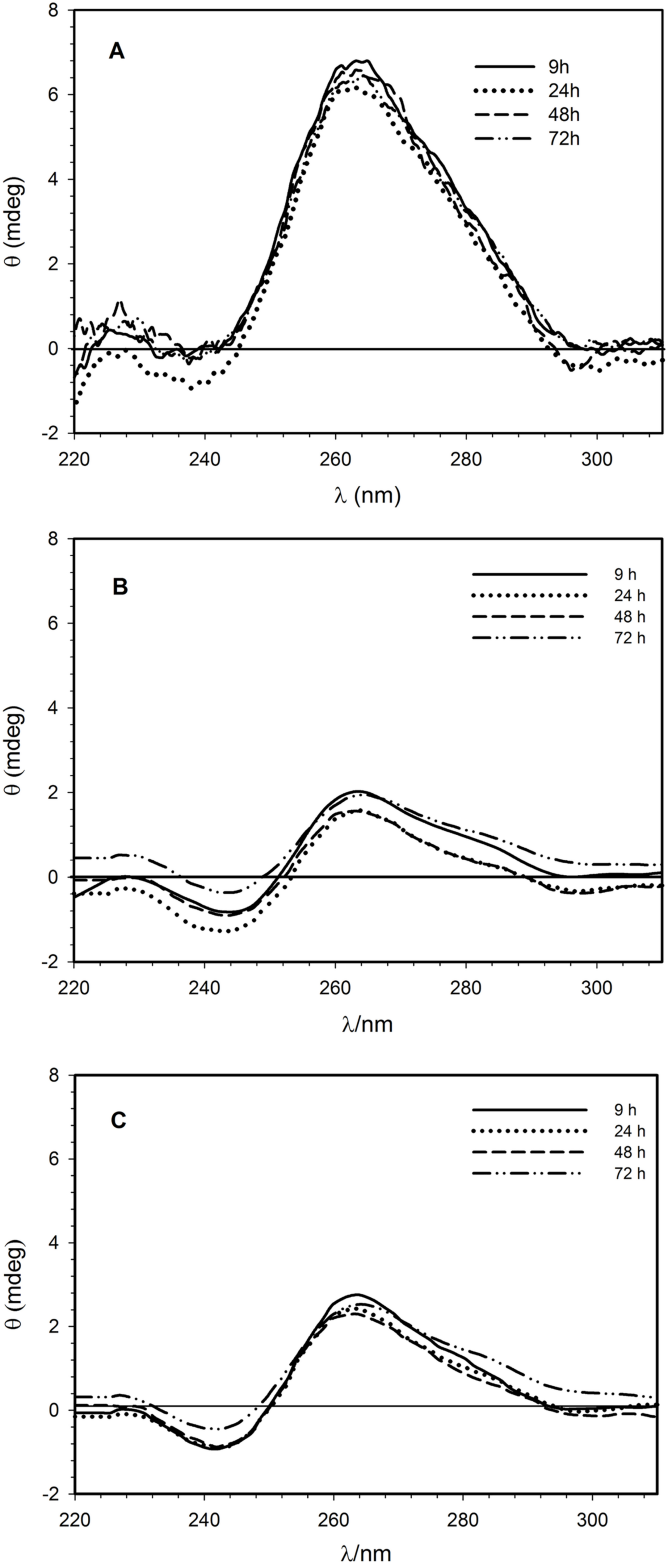
Circular dichroism spectroscopy. (A) CD spectra of 1.3 **μ**M siRNA in buffer solution (5 mM HEPES, 0.1 mM EDTA, pH 7.4). (B) CD spectra of 1.3 **μ**M siRNA in the DMPC/1 formulation. (C) CD spectra of 1.3 **μ**M siRNA in the DMPC/2 formulation. Spectra were recorded at 25°C.

CD spectroscopy was used to investigate interactions between siRNA and liposomes. Differently from the polymorphism of double-stranded DNA, RNA duplexes are conformationally confined to two closely related A and A’ structures [48]. Therefore, CD bands of RNA do not undergo strong changes. As expected, the CD spectra of siRNA in lipoplexes (DMPC/1/siRNA and DMPC/2/siRNA) measured at different times after the preparation (Fig 1*B* for DMPC/1/siRNA and Fig 1*C* for DMPC/2/siRNA lipoplexes), resembles that of free siRNA in buffer solution reported for comparison in Fig 1*A*. The electrostatic interactions with cationic liposomes reduce the intensity of both the bands at about 265 and 240 nm. These results suggest that the association between liposomes and siRNA causes prevalent changes in the base pair stacking without consistent structural modification of the backbone. Furthermore, the lower intensity of both the dichroic bands indicates a destabilizing effect on siRNA conformation as confirmed by the melting profile (data not shown) where a continuous decreasing of the ellipticity measured at 264 nm is recorded at increasing temperature before the melting.

### DLS measurements to evaluate size and polydispersion of liposomes and lipoplexes

**Fig 2:**
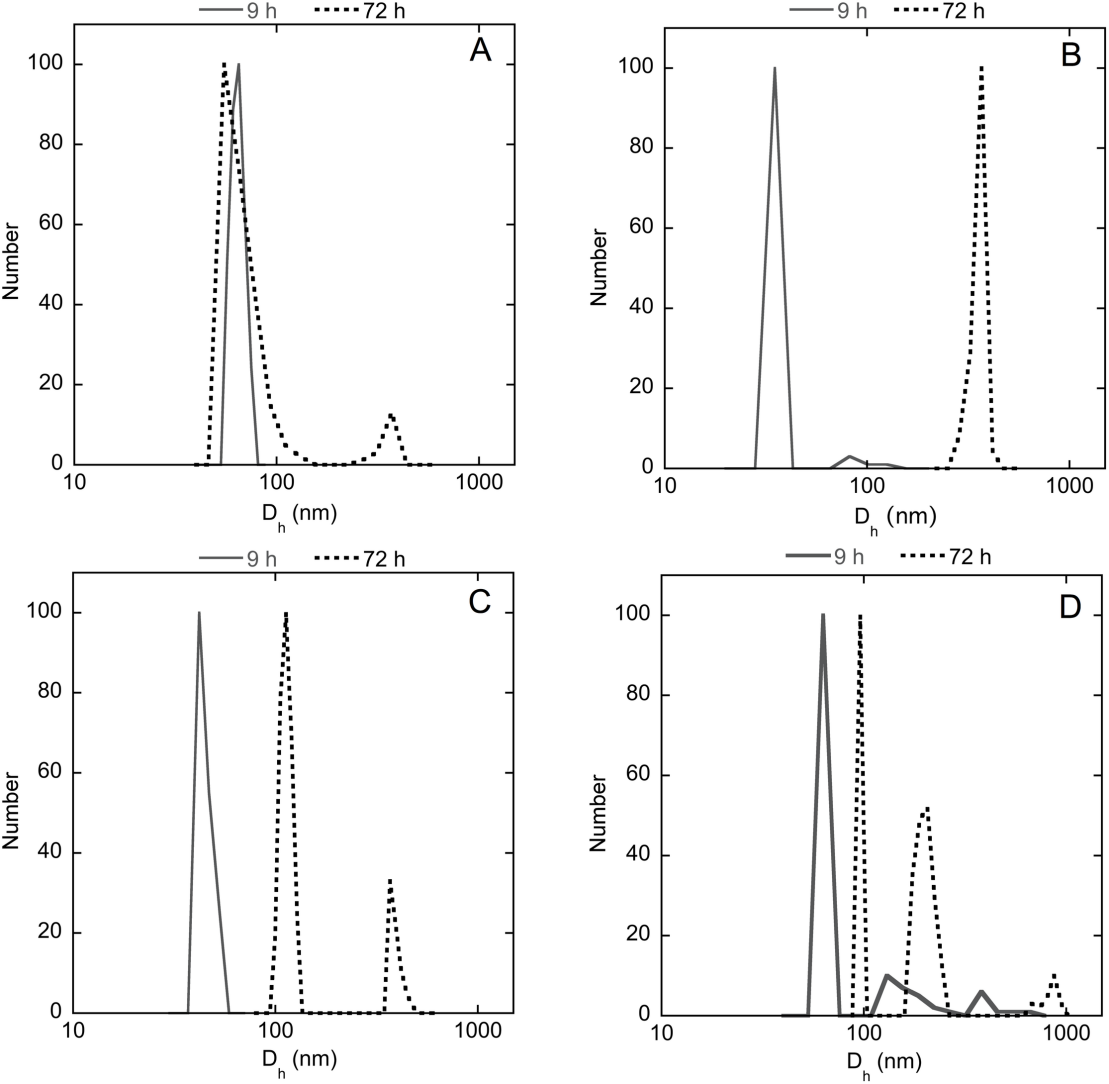
Dynamic light scattering. Hydrodynamic diameter distribution functions (averaged by number) obtained by CONTIN analysis of DLS data (scattering angle 90°, T = 25°C) for DMPC/1 liposomes (panel A); DMPC/2 liposomes (panel B); DMPC/1/siRNA lipoplexes (panel C); DMPC/2/siRNA lipoplexes (panel D).

The size distributions obtained by DLS analysis are reported as average by number. The CONTIN analysis of the autocorrelation function after 9 hours from the extrusion shows narrow single populations centered at ~60 and 40 nm, respectively for both DMPC/1 and DMPC/2 liposomes (Fig 2*A* and 2*B*, solid line). Liposome dimensions do not seem to be heavily affected by the presence of siRNA in the early stages, remaining in both cases under 100 nm in diameter diameter (42 and 63 nm, respectively, Fig 2*C* and 2*D*, solid line). After 72 hours from the preparation, the dimensions of DMPC/2 liposomes (Fig 2*B*, dot line) increase significantly whereas the dimensions of DMPC/1 liposomes (Fig 2*A*, dot line) remains almost unchanged, except for the appearance of a small population at ~700 nm in diameter. As regards the lipoplexes, the dimensions of both formulations DMPC/1/ siRNA and DMPC/2/siRNA do not change considerably, increasing slightly up to ~100 nm with the appearance of a less significant population at larger dimensions (Fig 2*C* and 2*D*). Such results suggest that the lipoplexes are rather stable; however, it is worth noting that the interaction between the lipoplexes and the membranes occurs after a few hours during the transfection process as shown by confocal microscopy experiments (*vide infra*).

### siRNAs are delivered in Jurkat T cells by a commercial transfection system

**Fig 3:**
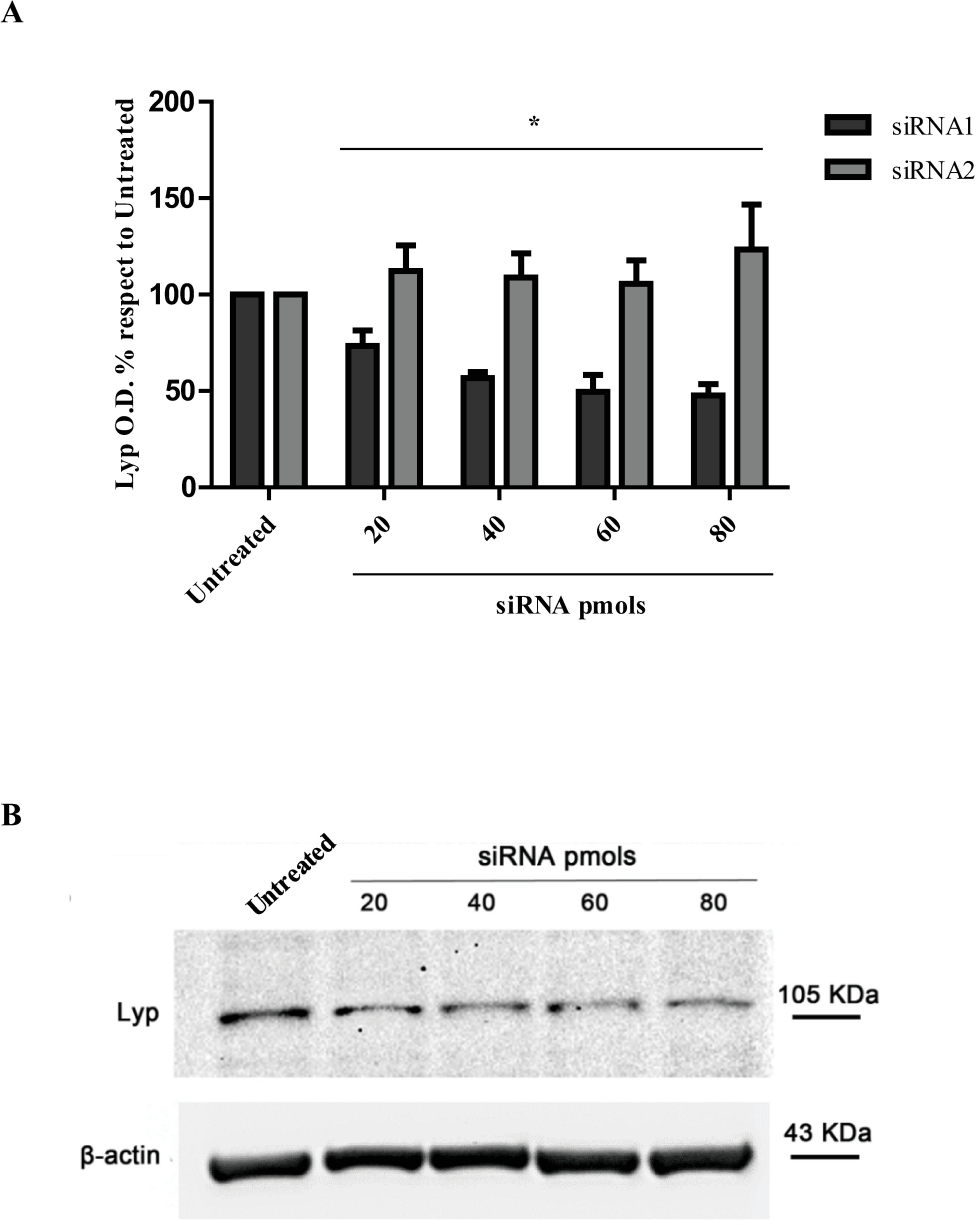
Lyp protein expression in Jurkat T cells after siRNA s/a transfection using a commercial system. (A) siRNA transfection with siRNA having higher affinity to the target mRNA sequence (siRNA1) resulted in a reduction of Lyp O.D. (optical density) percentages to untreated cells to 44%, 43% and 48% with the doses of 40-60-80 pmols respectively after 48 hours of transfection. O.D. values of Lyp and β-actin were obtained with ImageLab software. Lyp O.D. values for every treatment were normalized to the corresponding β-actin values. No reduction was obtained with siRNA molecule with lower affinity (siRNA2). * indicates p<0.05. (B) Representative WB image with all the corresponding experimental groups is shown. Lyp O.D. % with respect to untreated cells’ O.D. values calculated as percentage to untreated cells’ O.D. ‘Untreated’ represents the basal control level (100%) of Lyp protein expression in cells cultured in RPMI. The efficacy of transfection is calculated from the difference between 100 and the test O.D. percentage in each experiment.

In the experiment performed using the transfection kit to vehicle siRNA s/a duplex, having higher affinity for the target mRNA sequence (siRNA1), a dose/response reduction of Lyp protein expression (known to be a 5 hours half-life protein [49]) was obtained treating Jurkat T cells with 40–80 pmols of siRNA (Fig 3). In particular after 48 hours of transfection we observed a maximal 48% inhibition with 80 pmols of siRNA in respect to control untreated cells. The siRNA s/a molecule with lower affinity (siRNA2) showed no efficacy in respect to the ability of interfering with Lyp protein levels, so no decrease was observed compared to control samples, as to indicate the specificity of the siRNA molecule chosen for the subsequent experiments reported below (Fig 3 and S3 Fig).

### Jurkat T cells can be transfected with custom liposome formulations

#### Efficacy of DMPC/1/siRNA lipoplexes in Jurkat T cells

The results obtained employing DMPC/1/siRNA lipoplexes indicated their efficacy in reducing Lyp protein expression levels in Jurkat T cells already after 48 hours from the beginning of transfection. In particular we found that transfection was efficient with the same range of doses used in the commercial transfection system (Table 1).

**Table 1:** DMPC/1/siRNA treatment of Jurkat T cells, 48 hours from the beginning of transfection.

DMPC/1	Percentages of Lyp expression		
Treatment	60 minutes	90 minutes	O/N
			
RPMI	100	100	100
DMPC/1	104.5 ± 0.6	97.4 ± 0.6	102.7 ± 2.8
siRNA 20 pmols	105.6 ± 3.1	101.0 ± 0.3	98.8 ± 6.6
DMPC/1/siRNA 20 pmols	64.2 ± 0.2	93.3 ± 0.5	68.8 ± 6.5
			
RPMI	100	100	100
DMPC/1	102.9 ± 1.8	98.8 ± 2.9	97.3 ± 1.4
siRNA 60 pmols	104.9 ± 7.3	108.5 ± 6.1	95.0 ± 4.3
DMPC/1/siRNA 60 pmols	71.0 ± 6.8	72.3 ± 3.1	61.0 ± 1.5
			
RPMI	100	100	100
DMPC/1	103.7 ± 3.7	102.7 ± 0.2	100.4 ± 2.4
siRNA 100 pmols	107.2 ± 8.3	106.5 ± 7.0	102.6 ± 0.9
DMPC/1/siRNA 100 pmols	62.3 ± 3.3	76.4 ± 4.9	53.3 ± 3.7
			

Representative duplicate experiment (S4 Fig). Results are expressed as mean ± SD.

**Fig 4:**
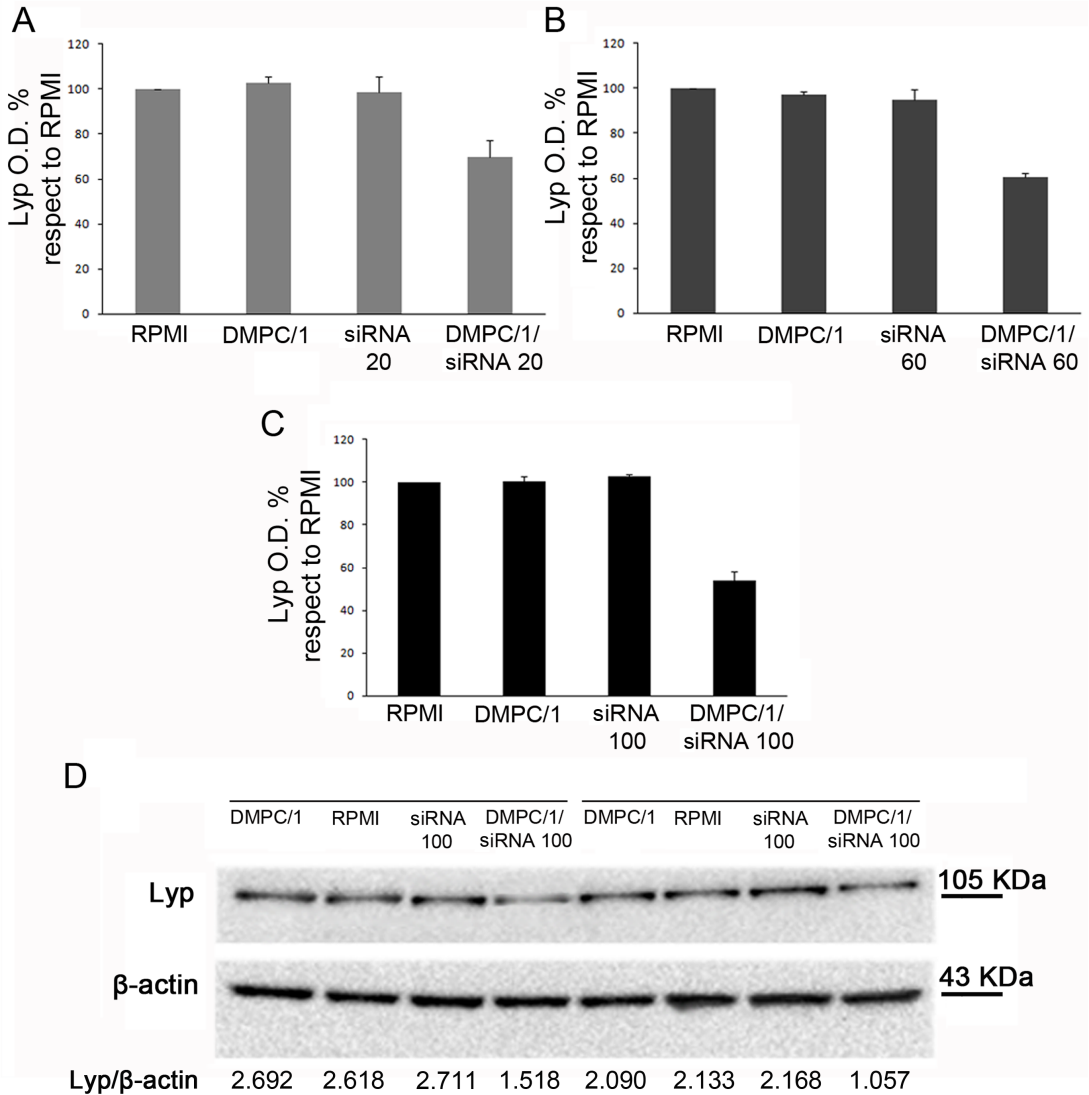
Lyp protein levels in Jurkat T cells 48 hours after the beginning of O/N transfection with different doses of siRNA s/a in DMPC/1/siRNA lipoplexes. Representative duplicate experiment among all replicas in S4 Fig. (A) Lyp expression in Jurkat T cells cultured in RPMI or after O/N transfection with DMPC/1, 20 pmols of siRNA and 20 pmols of siRNA in DMPC/1/siRNA lipoplexes (DMPC/1/siRNA20). 20 pmols of siRNA complexed with DMPC/1 resulted in a 31% reduction of Lyp expression. (B) Same experiment as in A using 60 pmols of siRNA complexed with DMPC/1 (DMPC/1/siRNA60). 39% reduction of Lyp expression was obtained. (C) Same experiment as in A using 100 pmols of siRNA complexed with DMPC/1 lipoplexes (DMPC/1/siRNA100). 47% reduction of Lyp expression was obtained. (D) Representative WB image within all experimental groups is shown. Under each blot Lyp O.D. values for every treatment are normalized over the corresponding β-actin values. All percentages were expressed relatively to untransfected cells (RPMI) that is considered the 100% of basal Lyp expression. Graphs A, B, C show the mean values and their standard deviations. In all experimental conditions (*vide infra*), a decrease in Lyp protein level was not observed in cells treated with the liposome alone or, more importantly, in cells treated with the correspondent dose of the siRNA s/a free molecule.

**Fig 5:**
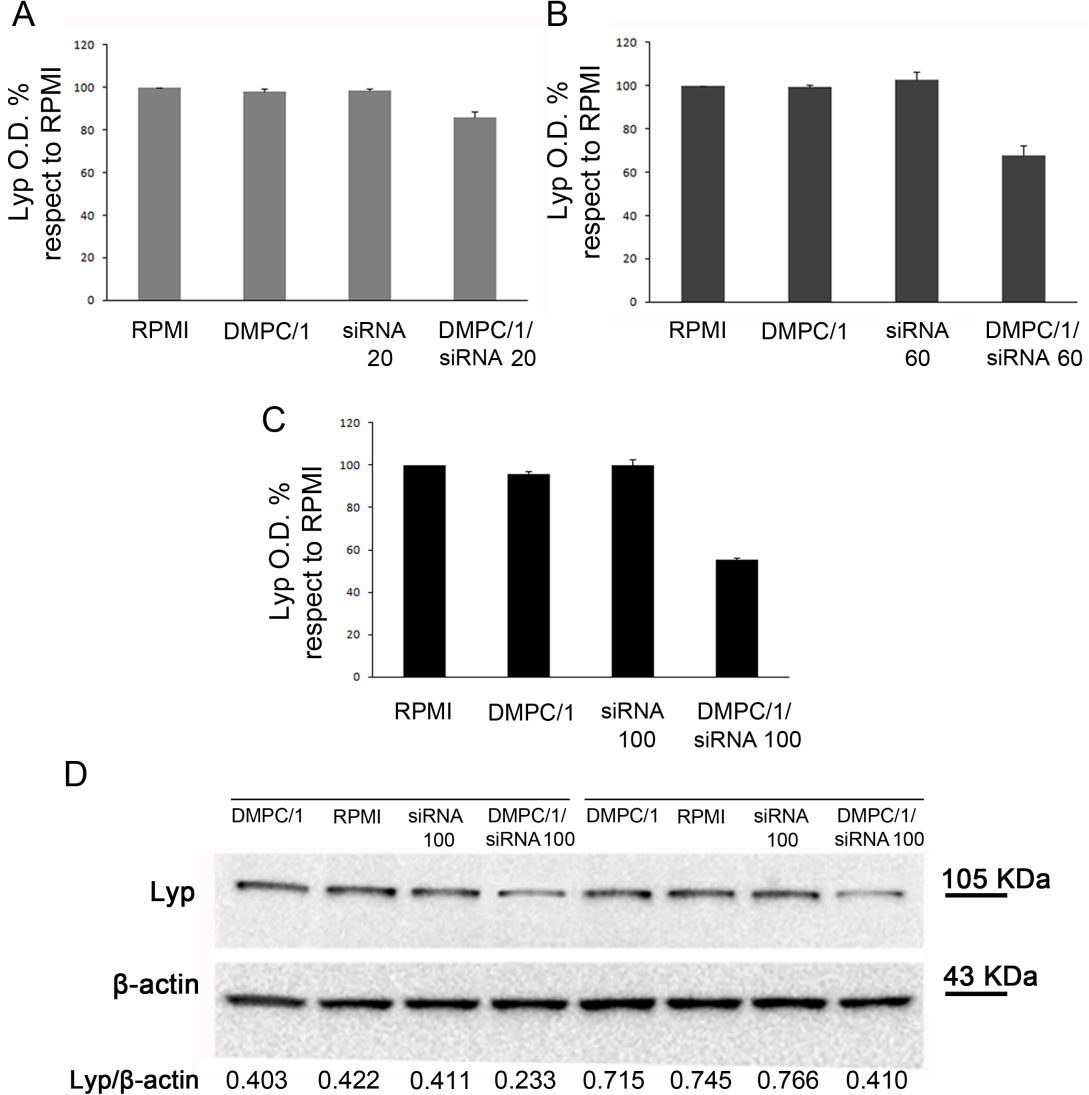
Lyp protein expression in Jurkat T cells 72 hours after the beginning of O/N transfection with different doses of siRNA s/a in DMPC/1/siRNA lipoplexes. Representative duplicate experiment among all replicas in S4 Fig. (A) Lyp expression in Jurkat T cells after O/N transfection with DMPC/1 alone (DMPC/1), 20 pmols of siRNA alone (siRNA20) and 20 pmols of siRNA complexed with DMPC/1 (DMPC/1/siRNA20) or cultured in RPMI. 20 pmols of siRNA complexed with DMPC/1 resulted in a 15% reduction of Lyp expression. (B) Same experiment as in A using 60 pmols of siRNA (siRNA60) complexed with DMPC/1 (DMPC/1/siRNA60). 33% reduction of Lyp expression was obtained. (C) Same experiment as in A using 100 pmols of siRNA (siRNA100) complexed with DMPC/1 (DMPC/1/siRNA100). 45% reduction of Lyp expression was obtained. (D) Representative WB image within all experimental groups is shown.

In a representative duplicate experiment administration of DMPC/1/siRNA lipoplexes for 60 minutes allowed until 36% reduction in the Lyp protein expression with respect to untreated cells using 20 pmols, 29% reduction with 60 pmols and 38% reduction with 100 pmols of siRNA (data not shown, Table 1). In cells treated with DMPC/1/siRNA lipoplexes O/N, we observed 31% reduction in Lyp protein level using 20 pmols, 39% reduction using 60 pmols and 47% reduction using 100 pmols of siRNA (Fig 4, Table 1). These data indicating the decrease of Lyp protein expression were also compared to the data relative to cells treated with empty liposomes, which, on the other hand, did not show any inhibition effect. Similar results of inhibition were obtained after 72 hours of transfection (Fig 5) (Table 2).

**Table 2:** DMPC/1/siRNA treatment of Jurkat T cells, 72 hours from the beginning of transfection.

DMPC/1	Percentages of Lyp expression		
Treatment	60 minutes	90 minutes	O/N
			
RPMI	100	100	100
DMPC/1	95.2 ± 1.0	100.5 ± 0.6	98.1 ± 1.3
siRNA 20 pmols	99.4 ± 6.0	98.2 ± 0.2	98.3 ± 0.9
DMPC/1/siRNA 20 pmols	71.4 ± 1.3	87.4 ± 6.1	85.2 ± 2.3
			
RPMI	100	100	100
DMPC/1	94.5 ± 0.2	97.3 ± 2.7	99.2 ± 1.0
siRNA 60 pmols	97.8 ± 2.2	96.4 ± 1.9	102.6 ± 4.0
DMPC/1/siRNA 60 pmols	55.7 ± 0.7	57.2 ± 3.2	67.3 ± 4.3
			
RPMI	100	100	100
DMPC/1	101.7 ± 3.2	100.7 ± 3.2	95.8 ± 0.2
siRNA 100 pmols	104.9 ± 0.2	103.4 ± 3.1	100.1 ± 2.7
DMPC/1/siRNA 100 pmols	61.6 ± 0.8	68.2 ± 0.5	54.7 ± 0.6
			

Representative duplicate experiment (S4 Fig). Results are expressed as mean ± SD.

Interestingly, in all experimental conditions the decrease in Lyp protein levels was not observed in cells treated with the liposomes alone and more importantly in cells treated with the correspondent doses of siRNA s/a not complexed with liposomes. Moreover, the reductions observed were consistent across biological replicas.

Statistical significance in the reduction of Lyp protein levels was revealed by the analysis of all biological replicas (S4 Fig).

#### Efficacy of DMPC/2/siRNA lipoplexes in Jurkat T cells

The results obtained with DMPC/2/siRNA lipoplexes highlighted its efficacy in reducing Lyp protein expression in Jurkat T cells (Tables 3 and 4). After 48 hours from the beginning of the 60 minutes transfection we observed 29–35% reduction in Lyp expression in Jurkat T cells treated with 20–100 pmols of siRNA (data not shown, Table 3). As previously observed for DMPC/1/siRNA, a more consistent reduction (23–51%) was achieved incubating cells for a longer period as indicated by the O/N transfection data (S5 Fig). After 72 hours of culture since the beginning of the transfection similar data of inhibition of Lyp protein expression were obtained with the same range of doses (Table 4). S6 Fig shows the statistical significance obtained from the analysis of all biological replicas performed using DMPC/2 lipoplexes.

**Table 3:** DMPC/2/siRNA treatment of Jurkat T cells, 48 hours from the beginning of transfection.

DMPC/2	Percentages of Lyp expression		
Treatment	60 minutes	90 minutes	O/N
			
RPMI	100	100	100
DMPC/2	104.4 ± 1.2	98.7 ± 2.5	97.9 ± 0.5
siRNA 20 pmols	99.9 ± 0,1	103.9 ± 3.0	103.0 ± 4.6
DMPC/2/siRNA 20 pmols	70.8 ± 1.1	90.4 ± 0,1	77.5 ± 1.6
			
RPMI	100	100	100
DMPC/2	100.9 ± 4.1	93.6 ± 0.3	101.9 ± 2.8
siRNA 60 pmols	99.2 ± 0.1	97.1 ± 2.6	98.6 ± 1.2
DMPC/2/siRNA 60 pmols	65.0 ± 1.5	63.1 ± 1.9	48.9 ± 0.7
			
RPMI	100	100	100
DMPC/2	100.8 ± 0.2	99.1 ± 0,2	98.6 ± 6.4
siRNA 100 pmols	101.9 ± 5.8	103.0 ± 3.2	95.2± 4.1
DMPC/2/siRNA 100 pmols	68.2 ± 4.2	67.7 ± 2.1	58.1 ± 4.5
			

Representative duplicate experiment (S6 Fig). Results are expressed as mean ± SD.

**Table 4:** DMPC/2/siRNA treatment of Jurkat T cells, 72 hours from the beginning of transfection

DMPC/2	Percentages of Lyp expression		
Treatment	60 minutes	90 minutes	O/N
			
RPMI	100	100	100
DMPC/2	101.1 ± 3.0	100 ± 1.9	100 ± 4.8
siRNA 20 pmols	108.5 ± 1.4	96.8 ± 0.1	103.6 ± 3.6
DMPC/2/siRNA 20 pmols	84.8 ± 2.6	95.7 ± 2.3	81.9 ± 3.6
			
RPMI	100	100	100
DMPC/2	105.3 ± 4.1	96.1 ± 1.0	104.5 ± 0.2
siRNA 60 pmols	106 ± 7.5	100.3 ± 2.0	99.5 ± 1.0
DMPC/2/siRNA 60 pmols	85.5 ± 0.9	69.2 ± 0.4	67.6 ± 1.7
			
RPMI	100	100	100
DMPC/2	98,6 ± 3.3	102.3 ± 4.3	93.0 ± 1.0
siRNA 100 pmols	99.4 ± 1.9	104.4 ± 3.7	102.4 ± 7.2
DMPC/2/siRNA 100 pmols	82.0 ± 0,8	66.5 ± 0.3	72.8 ± 1.2
			

Representative duplicate experiment (S6 Fig). Results are expressed as mean ± SD.

### PBMC are transfected with custom liposome formulations

The results obtained using the DMPC/1/siRNA incubation in HD PBMC after 48 hours of treatment did not show any variation of Lyp protein expression in these cells (S2*A* Table). On the other hand, after 72 hours culture since the beginning of the O/N transfection we observed a consistent reduction in the Lyp protein expression in the range of dose used (60–100 pmols of siRNA) (S2*B* Table). The major reduction however was obtained with the 60 pmols dose (S7 Fig). The reduction was 45% with 60 pmols, 25% with 80 pmols and 33% with 100 pmols of siRNA (S7 Fig and data not shown).

Transfection with the DMPC/2/siRNA in HD PBMC after both 48 hours and 72 hours provided a consistent 23–60% reduction in the Lyp protein expression in the range of dose used (60–100 pmols). The major decrease however was obtained with the 100 pmols dose of siRNA. This reduction was about 60% after 48 hours (S2*C* Table) and 52% after 72 hours (S2*D* Table). In detail after 48 hours from the beginning of the transfection, we obtained reductions of 35% (60 pmols), 37% (80 pmols) and 60% (100 pmols) (S2*C* Table). After 72 hours treatment, percentages of reduction were 23% (60 pmols), 29% (80 pmols) and 52% (100 pmols) (S8 Fig and S2*D* Table).

S9 Fig shows the statistical significance obtained from the analysis of all biological replicas performed using DMPC/2 lipoplexes.

### Confocal microscopy analysis

#### Lipoplexes incorporation is observed in Jurkat T cells and human PBMC

**Fig 6:**
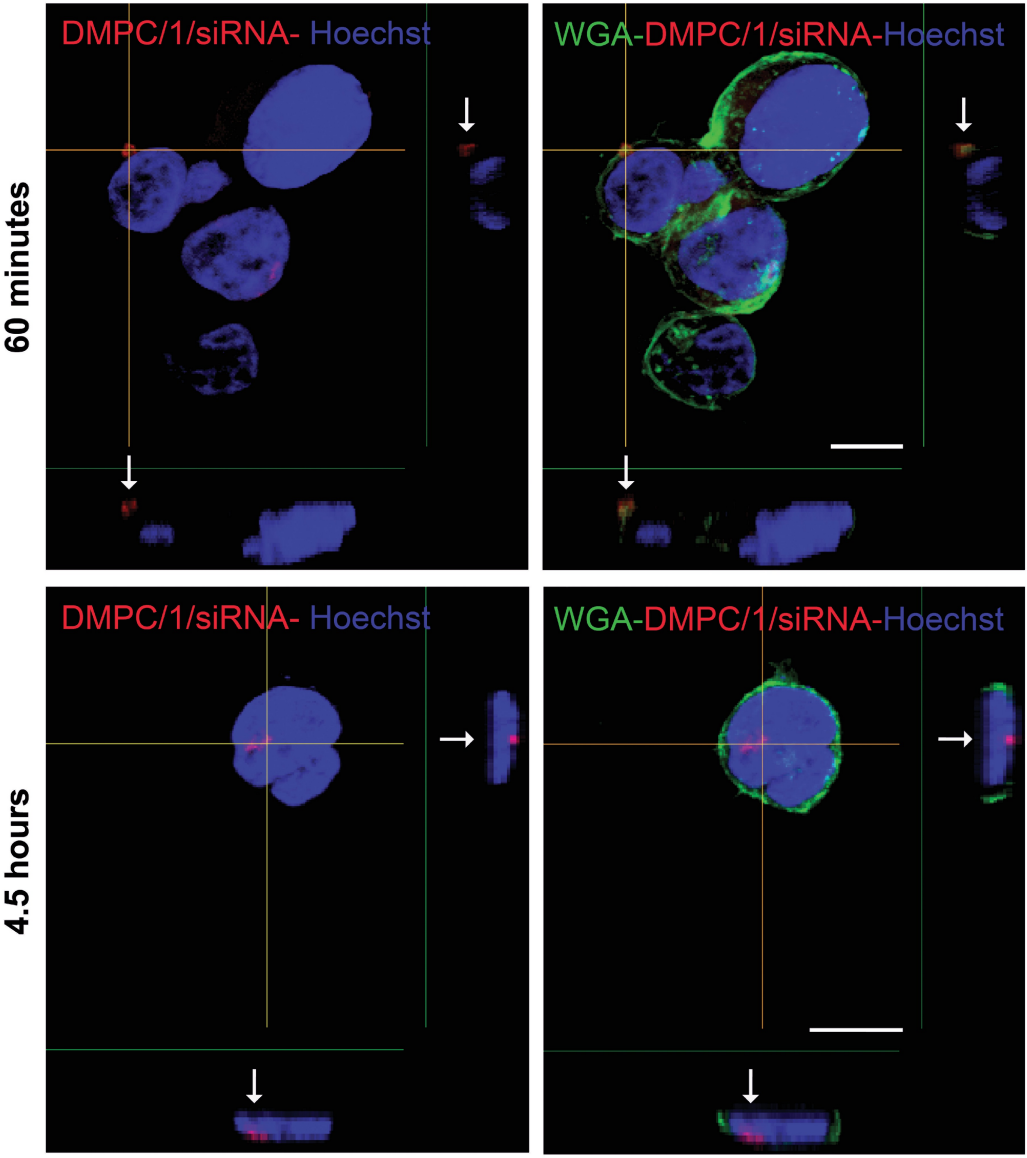
Confocal microscopy analysis of lipoplexes incorporation in Jurkat T cells. Data are shown after 60 minutes and 4 and half hours of incubation. Arrows indicate the localization of liposome fluorescence (red spots) compared to cell membrane (green) and nucleus (blue). Z-reconstructions obtained by confocal microscopy show the internalization of lipoplexes in Jurkat T cells in the X- and Y-axis projections. Plasma membrane is stained with wheat germ agglutinin (WGA), whereas nuclei are counterstained with Hoechst. Bar: 10 μm.

**Fig 7:**
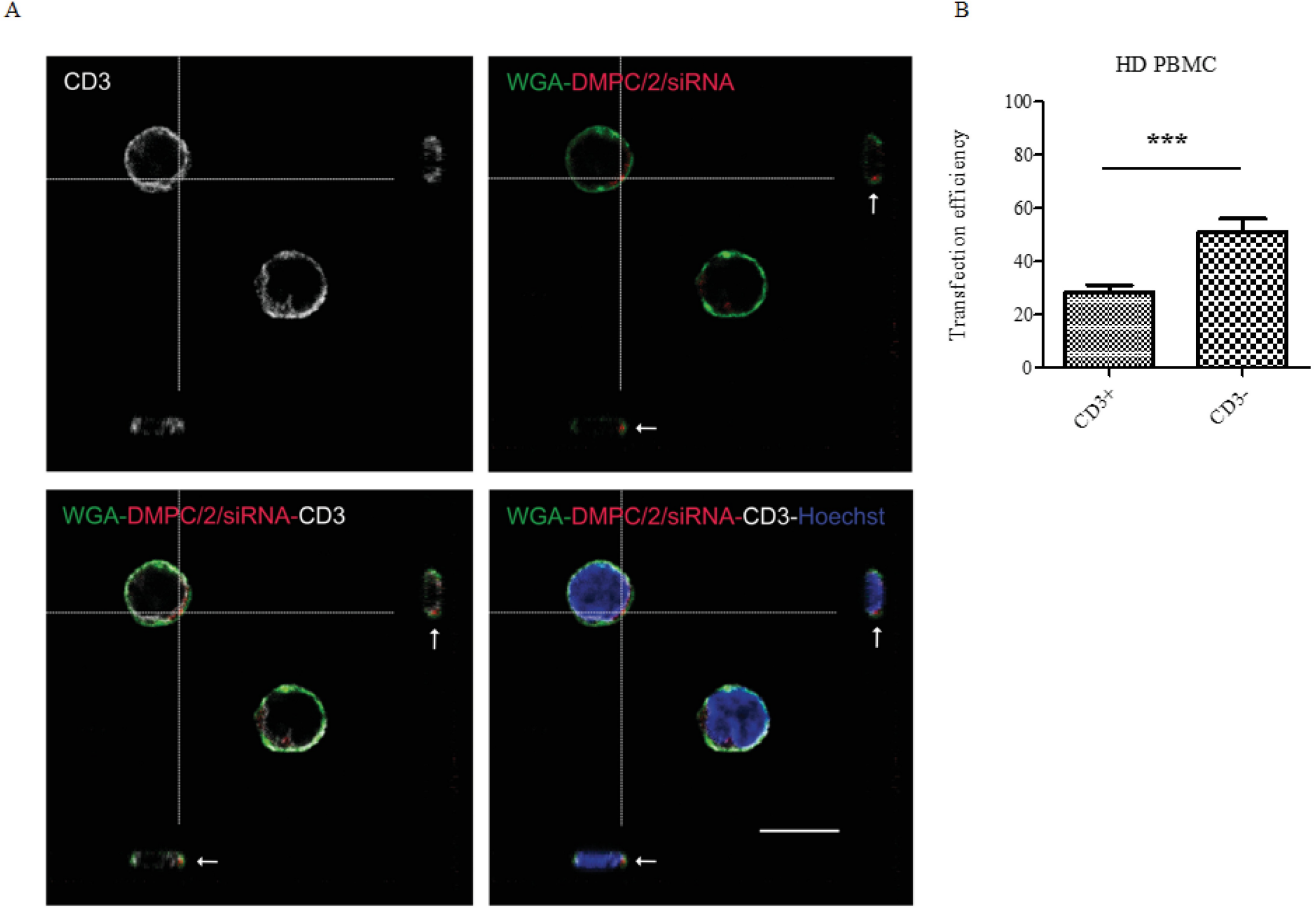
PBMC DMPC/2/siRNA internalization. HD PBMC were administered with DMPC/2/siRNA formulation marked with rhodamine. After 4 and a half hours of treatment cells were fixed and stained for immunofluorescence with anti-CD3 antibody (white) to specifically label T cells, WGA to stain plasma membrane (green), and Hoechst to label nuclei acid (blue). (A) Confocal Z reconstructions show the presence of the lipoplexes (red dots, arrows) in the cytoplasm of CD3^+^ cells. Bar: 10μm. (B) Graphs show the analysis of the percentage of siRNA^+^ cells among CD3^+^ and CD3^-^ cells by scoring a total number of 1546 cells. *** indicates a *p* value <0.001 (*p* = 0.0007).

After 4 and half hours of incubation with Jurkat T cells, the rhodamine-conjugated DMPC/1/siRNA lipoplexes (100 pmols of siRNA) were clearly visualized beneath the cell membranes (Fig 6, arrows) by the analysis of X- and Y-axis projections of Z-reconstructions of confocal single optical sections. The presence of rhodamine fluorescence inside the cells further indicates the efficacy of this delivery system in the internalization of siRNA molecules into Jurkat cells. After incubation with rhodamine-conjugated DMPC/2/siRNA lipoplexes (100 pmols of siRNA) (red dot) of human PBMC, internalization of the complexes was also specifically confirmed in T lymphocytes (CD3^+^ /white) (Fig 7*A*; S10 Fig), in B lymphocytes (CD19^+^/white) (S11*A and* S11*B* Fig), in antigen presenting cells (CD86^+^/white) (S11*A and* S11*B* Fig) and in the NK cells (CD56^+^/green) (S11*A and S*11*B* Fig). Statistical analysis of these data shows that the higher percentage of DMPC/2/siRNA positivity is found among the CD3^-^ when comparing CD3^+^ *versus* CD3^-^ cells (Fig 7*B*).

#### Lyp protein expression is detected in transfected Jurkat T cells and human PBMC

**Fig 8:**
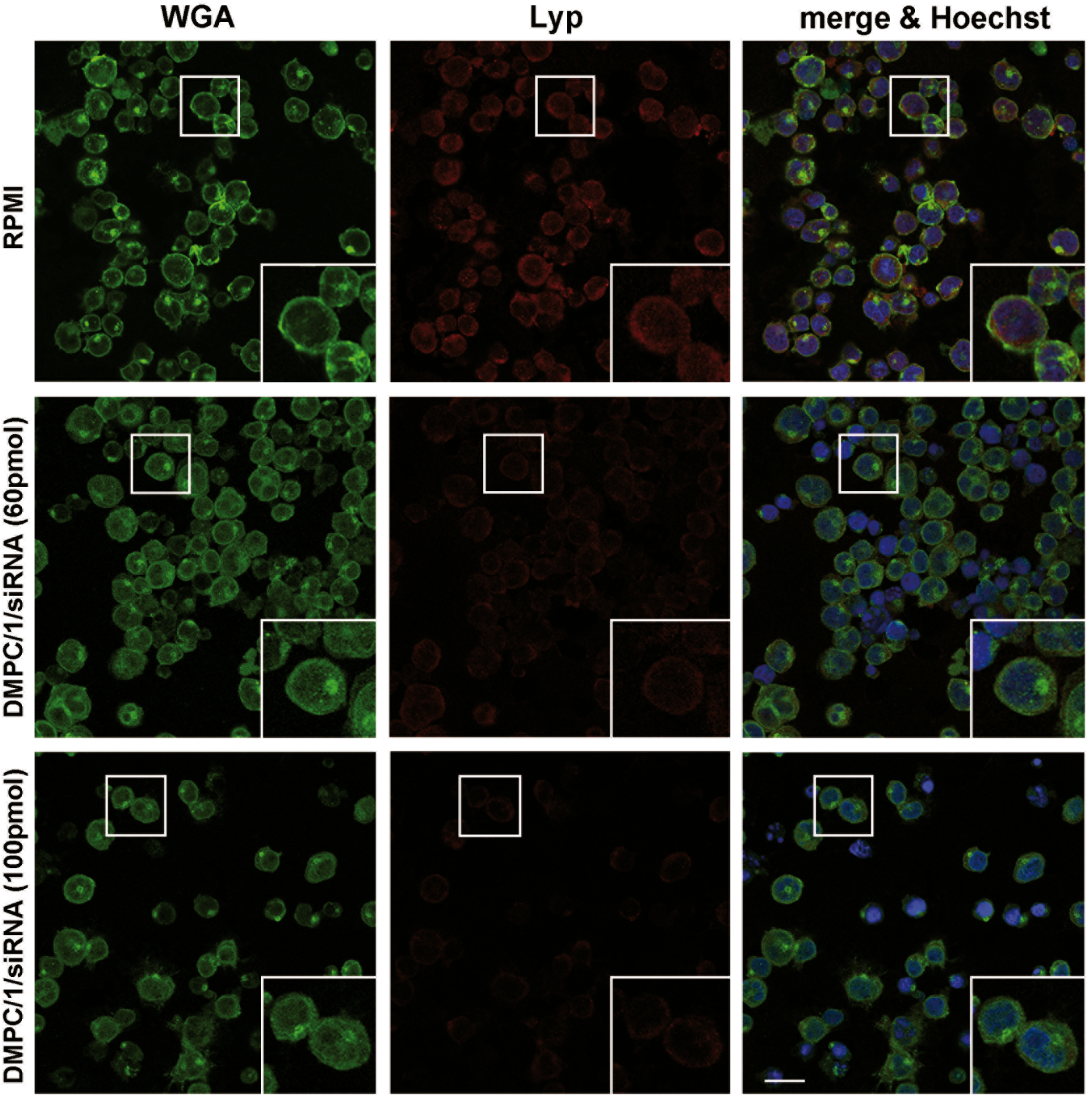
Confocal microscopy analysis of Lyp protein expression in Jurkat T cells after transfection with lipoplexes. The comparison shown is between samples transfected with 60 and 100 pmols of Lipo/siRNA lipoplexes, and analyzed after 72 hours from the beginning of the O/N transfection. Control cells are untreated and cultured in RPMI (upper panels). Lyp protein expression is revealed by anti-mouse IgG conjugated to Alexa Fluor 555 (red signal). Cell morphology and DNA are detected by WGA (green) and Hoechst (blue) staining, respectively. Bar: 20 μm.

**Fig 9:**
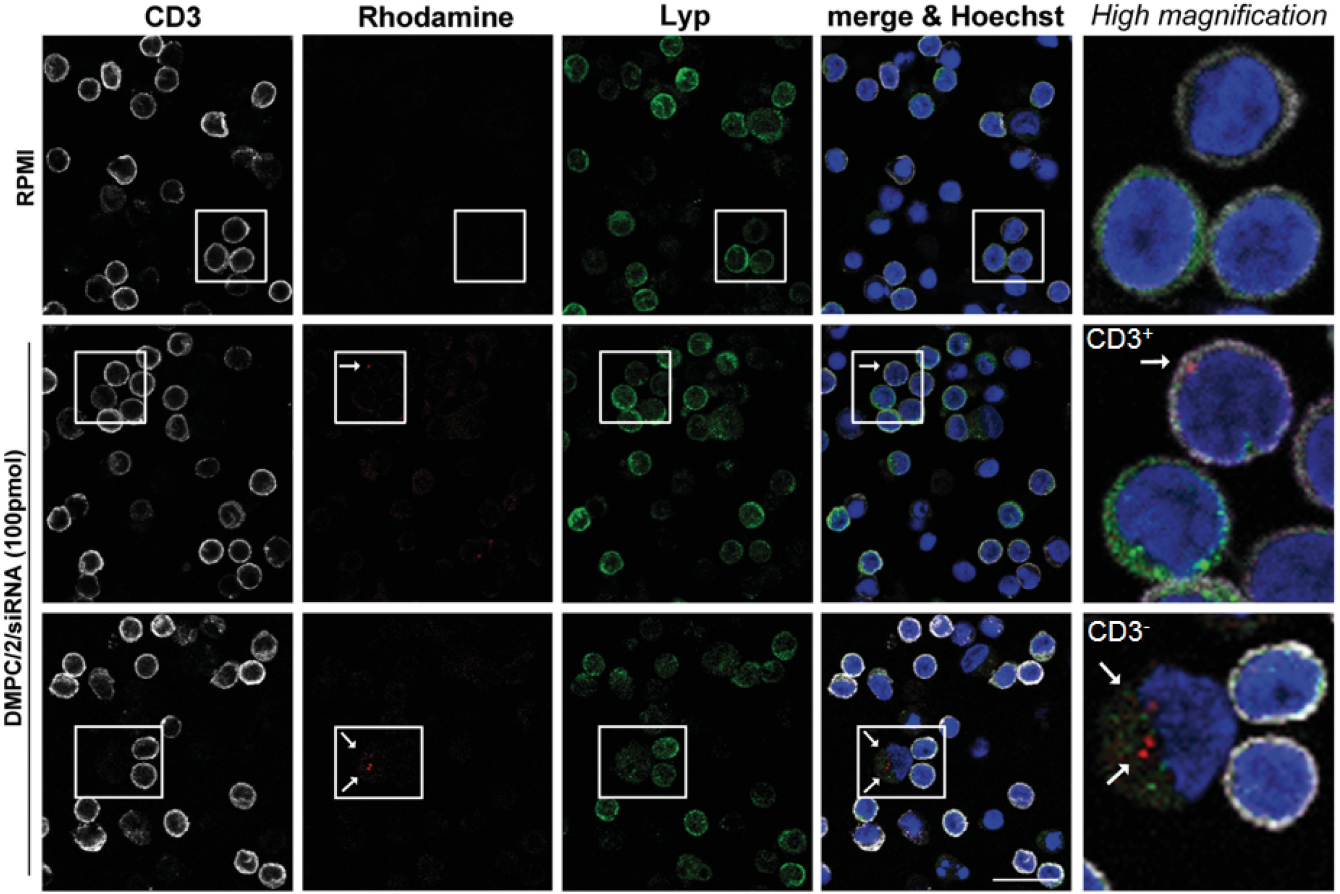
Confocal microscopy analysis of Lyp protein expression in human PBMC after transfection with lipoplexes. The images show the concurrence of the lipoplexes (red dot/arrow) inside CD3^+^ (white) and CD3^-^ cells and the reduction in Lyp immunofluorescence signal (green). Cells nuclei are counterstained with Hoechst dye (blue) in comparison with untransfected cells. Bar: 20 μm.

Immunofluorescence and confocal microscopy analysis of Lyp protein expression carried out in Jurkat T cells after 48 and 72 hours from the beginning of the O/N transfection, both revealed a significant reduction of the protein expression level (Fig 8). The same study, performed on human PBMC by administering the cells with rhodamine-conjugated lipoplexes (100 pmols of siRNA) (red dot/arrow in Fig 9), showed their efficacy to reduce Lyp protein levels following internalization in both CD3^+^ (white) and CD3^-^ cells (Fig 9).

### Lipoplexes are not toxic to human lymphocytes

Either cells treated with a commercial transfection system or lipoplexes did not show signs of toxicity during the culture period. Lipoplexes treatment did not affect Jurkat T cells proliferation (S3 Table).

Human PBMC treated with rhodamine-marked DMPC/2/siRNA (100 pmols) for 4 and a half hours retain a healthy looking morphology either of the cell membrane (green) and of the nuclei (blue) as revealed by confocal microscopy. These cell compartments did not show signs of damage or apoptosis respectively (S10 Fig).

**Fig 10:**
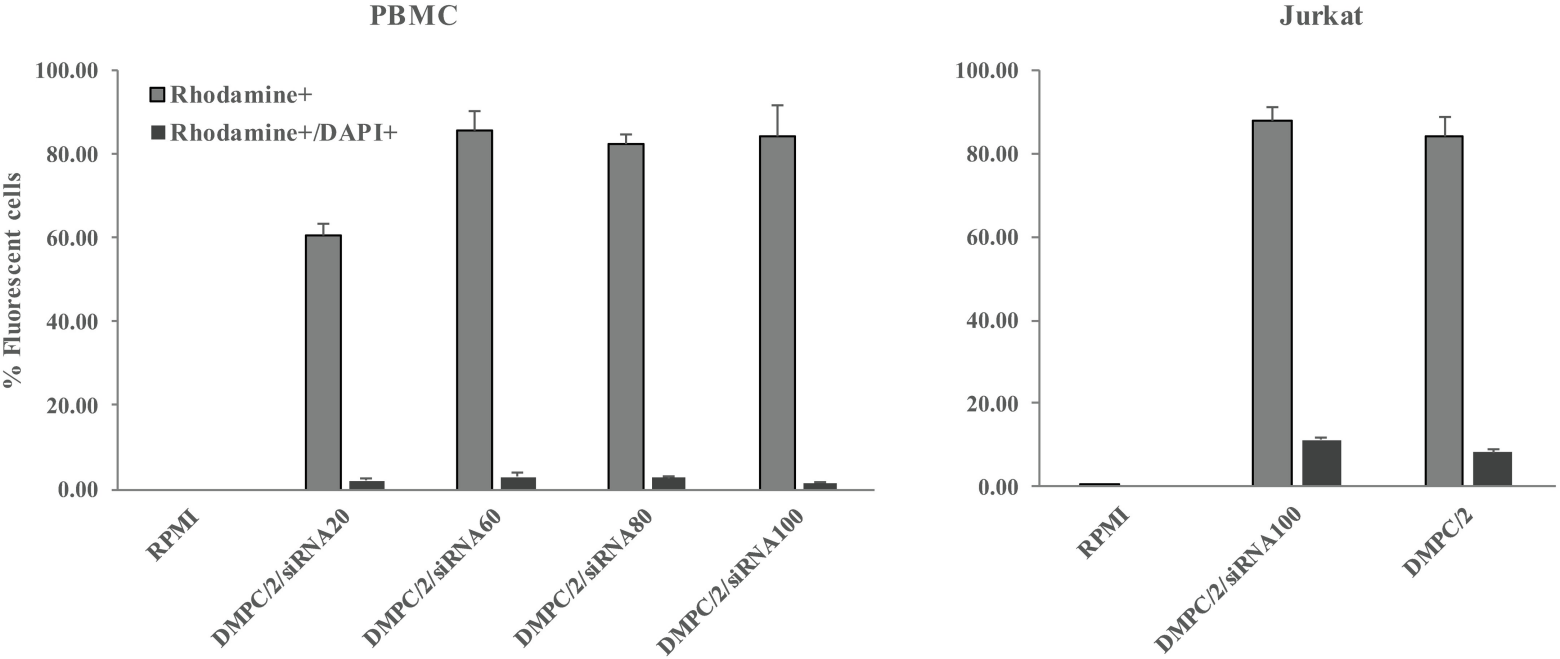
Evaluation of cell death after lipoplexes internalization. Flow cytometric analysis of HD PBMC (left) and Jurkat cells (right) treated with rhodamine-marked lipoplexes for 4 and a half hours. The histogram shows both the percentage of ‘transfected’ lymphocytes (rhodamine^+^ cells) and the percentage of dead cells among the transfected ones (DAPI^+^ and rhodamine ^+^ cells).

In Flow-cytometry analysis of both Jurkat T cells and PBMC, within the population of cells incorporating rhodamine-marked lipoplexes a low percentage of DAPI positive dead elements was detected indicative of absence of toxicity. These experiments also confirmed good internalization efficiency of siRNA complexes as revealed by percentages of rhodamine positive cells (Fig 10).

### Functional assays of the effect of lipoplexes’ treatment

Jurkat T cells transfected O/N with lipoplexes were stimulated using anti-CD3/CD28 beads to engage TCR signaling. In agreement with data already reported in literature [50] that a signal transmitted through the CD3 is by itself insufficient for activation of Jurkat T cells, undetectable levels of IL-2 were assayed in ELISA in basal conditions. Nevertheless, treatment with lipoplexes with the siRNA dose in the range of 60–100 pmols indeed induced significant IL-2 molecule production (S12 Fig).

**Fig 11:**
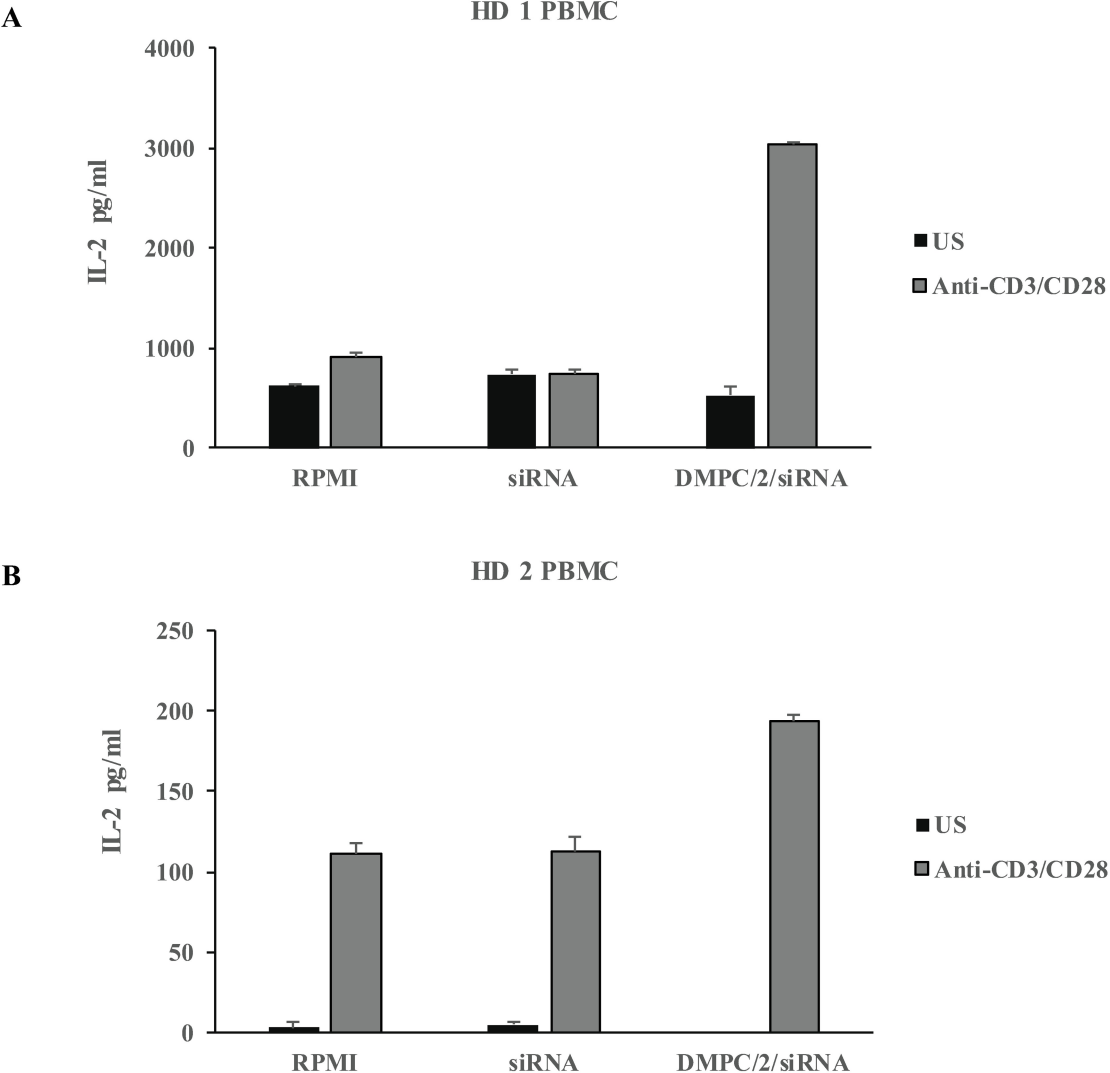
IL-2 detection ELISA assay of culture supernatants. Histograms illustrate the increment of IL-2 upon 5 days of anti-CD3/CD28 activation on PBMC derived from two healthy volunteers HD1 (A) and HD2 (B) previously transfected O/N with DMPC/2/siRNA60. The amount of IL-2 was normalized to the total protein load of each sample (estimated by Thermo Scientific BCA colorimetric protein assay kit). US = control unstimulated cells.

In supernatants of PBMC cultures where TCR signaling was engaged through anti-CD3/CD28 beads the effect of lipoplexes transfection was revealed by increased IL-2 production indicating impairment of Lyp activity (Fig 11).

In supernatants of Jurkat T cells stimulated using PMA-Ionomycin impaired IL-2 secretion as a result of lipoplexes transfection (S13 Fig). PMA-Ionomycin bypasses TCR signaling; it acts on a CSK-independent regulation of Lyp by enhancing protein kinase C (PKC)-mediated phosphorylation of Lyp-Ser-35 in the catalytic domain thereby inhibiting the phosphatase activity of the protein [13] and augmenting indirectly TCR activation. In the context of an already occurred inhibition of *PTPN22* the pathway is further deregulated, there is no T cell activation, therefore IL-2 secretion is critically impaired as observed in S13 Fig.

## Discussion

The development of new immunotherapies to induce cell tolerance has been an important challenge in the management of autoimmune endocrine conditions especially T1DM [9]. An ideal approach should be able to halt or delay the immune attack of the β cells, being devoid of systemic side effects, preserve immunity against infections and tumors and permit β cell regeneration. The treatment may help to improve the stability of the metabolic course of the disease thereby hampering or reducing long-term complications. Over the last decade, the promising results obtained from experiments performed using animal models led to the development of clinical trials in humans. However, most of the trials either using antigen-specific therapies, T and B lymphocytes targets, anti-inflammatory approaches, cytokines or stem cells failed to achieve insulin-independence in T1DM patients [51]. Furthermore whenever the results of Phase I and Phase II trials were promising, large randomized controlled trials did not reach primary endpoints [52,53]. The main problem resides on the difference between the human and the mouse model, the contribution of a plethora of environmental factors to the pathogenesis, inaccurate dosing, time/duration of treatment and last but not least the heterogeneity of the human disease influenced by different genetic determinants often requiring ‘tailored’ approaches of personalized medicine [54].

The allelic variant of *PTPN22* R620W is strongly associated with T1DM in humans increasing the risk of disease by 2–4 fold [reviewed in 55]. The most definitive evidence of enhanced penetrance of autoimmune diabetes, with earlier onset and increased insulin-autoantibodies was recently produced in non-obese diabetic mice (NOD) by introducing the mouse orthologue *PTPN22*^*R619W*^ [55]. The effect of the variant is still debated in the literature with studies generally supporting a ‘gain of function’ model since phosphatase with a higher catalytic activity is produced causing a more potent regulation of T cell activation [14]. In addition, studies more recently produced demonstrated the effect on regulatory T cells (Tregs). As regard Maine et al (2012) [56] demonstrated in knockout mice alterations of the peripheral Tregs while increasing their thymic selection. Other studies [57,58] also report a ‘gain of function’ model of Tregs selection although *PTPN22* knockout indeed caused reduced TCR signaling. Overall, we have also to account for possible effects on T cell subgroups other than Tregs or additional effects on the immune system [59]. Further in the ‘gain of function’ model an increase in T cell activity may occur through loss of self-tolerance of peripheral T cells. The ‘loss of function’ model implies that a Lyp degradation is associated with lymphocyte and dendritic cell hyper responsiveness [60]. In the last model, putatively the loss of self- tolerance occurs earlier in the T cell life to be subsequently activated by autoantigens [59]. Whatever model is adopted in supporting the pathogenetic effect of the variant this can be a valid target for treatment throughout its down-modulation/knockdown in T1DM and APS3v patients by anyway restoring the net effect of the normal allele.

In an attempt to exploit a novel tailored immunotherapeutic approach, Delogu et al. [20] have already demonstrated that ASO to *PTPN22* can efficiently knockdown the gene by their delivery into Jurkat T cells using PNT that were shown of limited toxicity in mice [30]. In the light of the foregoing, we exploited the possibility of modulating the expression of the *PTPN22* gene in the Jurkat lymphoblastoid T cell line and in human PBMC cells by using original siRNA duplexes delivered by liposomal formulations known to be of limited toxicity in humans and therefore approved for human use by FDA as an alternative to PNT.

Nanoparticles with dimensions smaller than 100 nm in diameter have the potential to influence biological processes [61]. Their physico-chemical properties can have impact on their biodistribution, tropism and toxicity. Independently from the outcome of the experimental studies, the potential utility of PNT in humans have raised concerns being of low biodegradability and having a fibrous structure with potential toxic effects [62]. In particular it was clearly shown their capacity to induce systemic immunosuppression through the release of cytokines and prostaglandins [63]. As opposed to PNT, the use of liposomes revealed to be clinically feasible (*vide supra*) as delivery system for vaccine and cancer treatment and also for immunotherapies in autoimmune disorders [64,65]. In particular the use of auto antigen-loaded phosphatidylserine-liposomes was capable of arresting the autoimmunity process when administered to the non-obese diabetic (NOD) mice [66].

In our experiments, as expected, no signs of toxicity were observed in cultures of Jurkat T cells or PBMC. Transfection of both lipoplexes resulted in a significant reduction of Lyp protein expression in Jurkat T cells in the same range of doses used with the commercial transfection system. The reduction was estimated both in WB assay and by confocal microscopy analysis. In preliminary experiments, Liposome/siRNA complexes proved also to be efficient in affecting the Lyp protein expression in human PBMC as evaluated by WB analysis.

In preliminary experiments, we attempted to evaluate the biological consequences of *PTPN22* down-modulation in functional assays. We aimed to properly engage TCR signaling with anti-CD3/CD28 stimulation. In agreement with data already reported in literature [50] that a signal transmitted through the CD3 is by itself insufficient for activation of Jurkat T cells, undetectable levels of IL-2 were assayed in ELISA in basal conditions. Nevertheless, treatment with lipoplexes with the siRNA dose in the range of 60–100 pmols indeed induced significant IL-2 molecule production (S12 Fig). Increase of IL-2 secretion was similarly obtained in culture supernatants of PBMC following anti-CD3/CD28 TCR-driven stimulation (Fig 11) clearly indicating the impairment of Lyp inhibitory activity.

We also demonstrated that transfection with lipoplexes impaired IL-2 secretion from Jurkat T cells following PMA/IONO stimulation. This acts on a CSK-independent regulation of Lyp by enhancing protein kinase C (PKC)-mediated phosphorylation of Lyp-Ser-35 in the catalytic domain thereby inhibiting the phosphatase activity of the protein [13] thus augmenting indirectly TCR activation. On a speculative basis in our specific case *PTPN22* would be inhibited and consequently the pathway already deregulated, thus T cell activation is not expected and IL-2 secretion is impaired. We need however to point out that interference of lipoplexes with other mechanisms could contribute to the observed phenomenon. To our knowledge, to date, the structure and biochemical pathways that directly regulate Lyp function are not completely elucidated. Indeed Lyp task is accomplished by the dephosphorylation of several kinases including LCK, Fyn and ZAP-70 (rev in [67]) and is effective when dissociated from CSK [68]. Therefore in this set of experiments carried out with PMA/IONO the mechanistic insights, leading to impaired IL-2 production, would require further unravelling before being clearly explained. On a speculative basis, addition of lipoplexes might favour the inhibitory effect of free CSK on LCK activation in absence of TCR engagement [68].

The two formulations gave rise to similar results. Differently from previous results in which the stereochemistry of the spacer of the gemini was found to strongly influence the delivery efficiency of the photosensitizer m-tetrahydroxyphenylchlorin to cell models of malignant glioma [41], in this case the stereochemistry of the two gemini surfactants 1 and 2 does not affect significantly the transfection properties of the lipoplexes.

Confocal microscopy analysis verified that lipoplexes were internalized in Jurkat T cells and HD PBMC after 4 and half hours of incubation. Previous studies have shown the possibility to increase the cell-type specific and cytoplasmic targeting of PEGylated carbon nanotubes both in ‘*in vitro*’ and in animals [29]. An intracellular delivery approach was unraveled to improve targeting of specific cell-surface receptors, the internalization of nanoassemblies *via* the endosome/lysosome system and disruption of the lysosomal compartment [29]. We can envisage that, in developing new therapeutics, the intracytoplasmic T cell specific delivery within PBMC could be enhanced using similar strategies based on functionalization of lipoplexes with FDA approved anti-CD3 mAbs [69].

We need however to underline that the delivery system was effective not only on CD3^+^ but also in CD3^-^ PBMC. We could observe in confocal imaging that in addition CD19^+^, CD56^+^ and CD86^+^ cells could be transfected. Consistently, reduction of Lyp expression was visualized in transfected CD3^+^ and CD3^-^ cells. These results suggest that the new treatment can have effect not only on T lymphocytes. Therefore it is possible to speculate that in future translational studies, aiming to target the variant allele, lipoplexes could produce different effects on different cells [56,60] therefore, as above discussed (*vide supra*) the ideal treatment would be the modulation/knockdown of the variant allele in any immunocyte to restore the net effect of the normal allele.

As final remark, our ‘*in vitro*’ data of efficacy and safety of lipoplexes on Jurkat T cells and PBMC await confirmation by ‘*in vivo*’ animal studies. These are mandatory before any translational approach could be attempted in humans. Nevertheless these preliminary data address the possibility of producing variant *PTPN22* allele selective inhibition by using short interfering RNA duplexes [70] as a novel immunotherapeutic approach to T1DM. The results of our strategy clearly envisage the possibility of *PTPN22* gene targeting by siRNA oligomers delivered to lymphocytes with liposomes, already utilized in clinical trials, for their minimal toxicity and biodegradability profiles.

## Supporting information

S1 Fig Chemical structure of liposomal formulations. (TIF)

S2 Fig Cell line authentication results. Genetic characteristics were determined by PCR-single-locus-technology on genomic DNA derived from Jurkat cells used for this study. Twenty-one independent PCR-systems were investigated (Eurofins Medigenomix Forensik GmbH, Ebersberg, Germany). Obtained data were searched and compared with online databases of human cell line short tandem repeats (STR) profiles (ATCC and DSMZ STR profile databases) and resulted in 100% match with Jurkat, Clone E6-1 Acute T Cell Leukemia Human.
(TIF)

S3 Fig Additional WB image. Results of siRNA1 transfection of Jurkat T cells with the commercial transfection system.
(TIF)

S4 Fig Analysis of Lyp protein expression in Jurkat T cells 48 and 72 hours after each transfection period with different doses of siRNA s/a complexed in DMPC/1/siRNA lipoplexes. Graphs show the analysis of Lyp protein expression 48 (upper panel) and 72 hours (lower panel) after the three different transfection periods in all control and lipoplexes treated groups. *** indicates a significant statistical difference (*p*<0.05) between lipoplexes treated Jurkat T cells and the control groups (DMPC/1; RPMI; siRNA100). DMPC/1: n = 9; RPMI: n = 7; siRNA100: n = 5; DMPC/1/siRNA20: n = 5; DMPC/1/siRNA60: n = 5; DMPC/1/siRNA100: n = 5.
(TIF)

S5 Fig Lyp protein expression in Jurkat T cells 48 hours after the beginning of O/N transfection with different doses of siRNA s/a in DMPC/2/siRNA lipoplexes. (A) Lyp expression in Jurkat T cells cultured in RPMI or after O/N transfection with DMPC/2, 20 pmols of siRNA (siRNA20) and 20 pmols of siRNA complexed in DMPC/2/siRNA lipoplexes (DMPC/2/siRNA20). 20 pmols of siRNA in DMPC/2/siRNA lipoplexes resulted in a 23% reduction of Lyp expression. (B) Same experiment as in A using 60 pmols of siRNA in DMPC/2/siRNA lipoplexes (DMPC/2/siRNA60). A 51% reduction of Lyp expression was obtained. (C) Same experiment as in A using 100 pmols of siRNA s/a in DMPC/2/siRNA (DMPC/2/siRNA100). A 42% reduction of Lyp expression was obtained. (D) Representative WB image with all experimental groups in two biological replicas is shown. Lyp O.D. values for every treatment were normalized with the corresponding β-actin values. All percentages were expressed relatively to untransfected cells (RPMI) that is considered the 100% of basal Lyp expression. Graphs A, B, C show the mean values and their standard deviations.
(TIF)

S6 Fig Analysis of Lyp protein expression in Jurkat T cells 48 and 72 hours after each transfection period with different doses of siRNA s/a complexed in DMPC/2/siRNA lipoplexes. Lyp protein expression 48 (upper panel) and 72 hours (lower panel) after transfection periods in all control and lipoplexes treated groups. DMPC/2: n = 8; RPMI: n = 7; siRNA100: n = 4; DMPC/2/siRNA20: n = 4; DMPC/2/siRNA60: n = 4; DMPC/2/siRNA100: n = 4.
(TIF)

S7 Fig Lyp protein expression in a healthy volunteer PBMC 72 hours after the beginning of O/N transfection with different doses of siRNA s/a in DMPC/1/siRNA lipoplexes. Figure shows representative WB image with all experimental groups. Cells were transfected with empty liposome, 100 pmols of siRNA alone, DMPC/1/siRNA 60, 80 and 100 pmols or cultured in RPMI.
(TIF)

S8 Fig Lyp protein expression in a healthy donor PBMC 72 hours after the beginning of O/N transfection with different doses of siRNA s/a in DMPC/2/siRNA lipoplexes. (TIF)

S9 Fig Analysis of Lyp protein expression in PBMC 48 and 72 hours after each transfection period with different doses of siRNA s/a complexed in DMPC/2/siRNA lipoplexes. Lyp protein expression 48 and 72 hours after the O/N transfection period in all control and lipoplexes treated groups. DMPC/2: n = 7; RPMI: n = 7; siRNA100: n = 3; DMPC/2/siRNA60: n = 3; DMPC/2/siRNA80: n = 3; DMPC/2/siRNA100: n = 3.
(TIF)

S10 Fig Confocal microscopy analysis of HD PBMC. HD PBMC were administered with the indicated formulation marked with rhodamine and relative controls. After 4 and a half hours of treatment cells were fixed and stained for immunofluorescence: anti-CD3 (Cy5/white) antibody to specifically label T cells, plasma membrane (WGA /green), and nuclei (Hoechst /blue) stains were used. Arrows indicate lipoplexes incorporation (dot spots). High magnification was shown for an improved visualization of dot internalization. Bar: 20 μm.
(TIF)

S11 Fig PBMC DMPC/2/siRNA internalization. HD PBMC were administered with the indicated formulation marked with rhodamine and relative controls. After 4 and a half hours of treatment cells were fixed and stained for confocal microscopy. (A) Three stainings were performed on PBMC by using: FITC anti human CD3 (green) antibody to specifically label T cells, and Alexa Fluor 700 anti-human CD19 (white) to mark B cells; FITC anti human CD3 (green) antibody and APC anti human CD86 (white) to detect antigen presenting cells; Cy5 anti human CD3 (white) and FITC anti human CD56 (green) to stain NK cells. Cell nuclei were counterstained with Hoechst (blue). Arrows indicate lipoplexes incorporation (red dot spots). Bar: 20 μm. High magnification was shown for an improved visualization of dot internalization. Bar: 10 mm. (B) The histograms indicate the transfection efficiency through percentage of siRNA^+^ cells among the indicated subsets analyzed. The separate histograms represent the mean for each population analyzed and label the standard deviation.
(TIF)

S12 Fig IL-2 detection ELISA assay of culture supernatants. Histogram shows the increase in IL-2 production from anti-CD3/CD28 beads treated Jurkat cells specifically after DMPC/2/siRNA administration at different doses. ^*^ indicates p<0.05.
(TIF)

S13 Fig IL-2 detection ELISA assay of culture supernatants. Histograms illustrate the unresponsiveness of IL-2 upon 24 (A, C) or 48 (B, D) hours of PMA/IONO (PMA-Ionomycin) stimulation in Jurkat T cells previously transfected with DMPC/2 liposomal formulation. US = control unstimulated cells; H = hours; PMA/IONO = transfected Jurkat T cells following activation with PMA/IONO. ** indicates p<0.01
(TIF)

S1 Table Design of siRNA duplexes to *PTPN22*. Table reports the design of duplex siRNAs SNP_C (s and a) against the wild type PTPN22 allele. SNPs were designed by genetists at Sigma Chemical Co.
(DOCX)

S2 Table DMPC/1/siRNA and DMPC/2/siRNA treatment of PBMC. Results obtained after 48 (A, C) and 72 hours (B, D) from the beginning of the transfection are listed.
(DOCX)

S3 Table Proliferative assay of DMPC/2/siRNA Jurkat T cells. Values of median fluorescence intensity (MFI) indicative of proliferation obtained from CMFDA-labelled Jurkat T cells after 48 and 72 hours from the beginning of transfection with DMPC/2 lipoplexes respect to untransfected cells.
(DOCX)
