# Supplementary figures and images for: Use of short interfering RNA delivered by cationic liposomes to enable efficient down-regulation of PTPN22 gene in human T lymphocytes
Source: PLoS One. 2017 Apr 24;12(4):e0175784. doi: 10.1371/journal.pone.0175784 (PMC5402975; doi:10.1371/journal.pone.0175784)

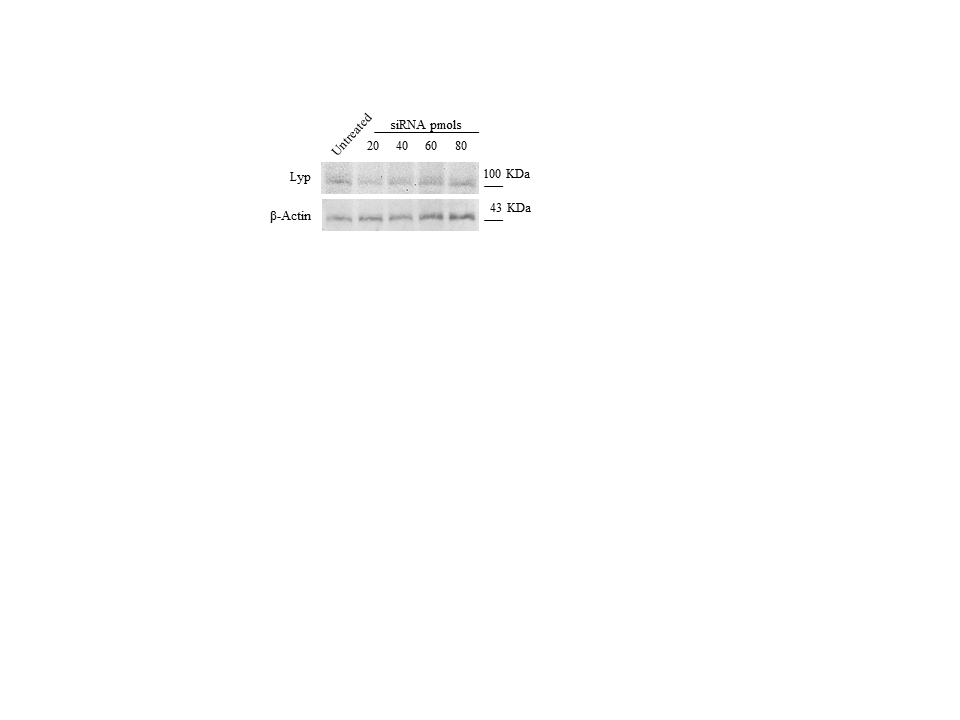

Supplement: S3 Fig — Results of siRNA1 transfection of Jurkat T cells with the commercial transfection system. (TIF) [file pone.0175784.s003.TIF]

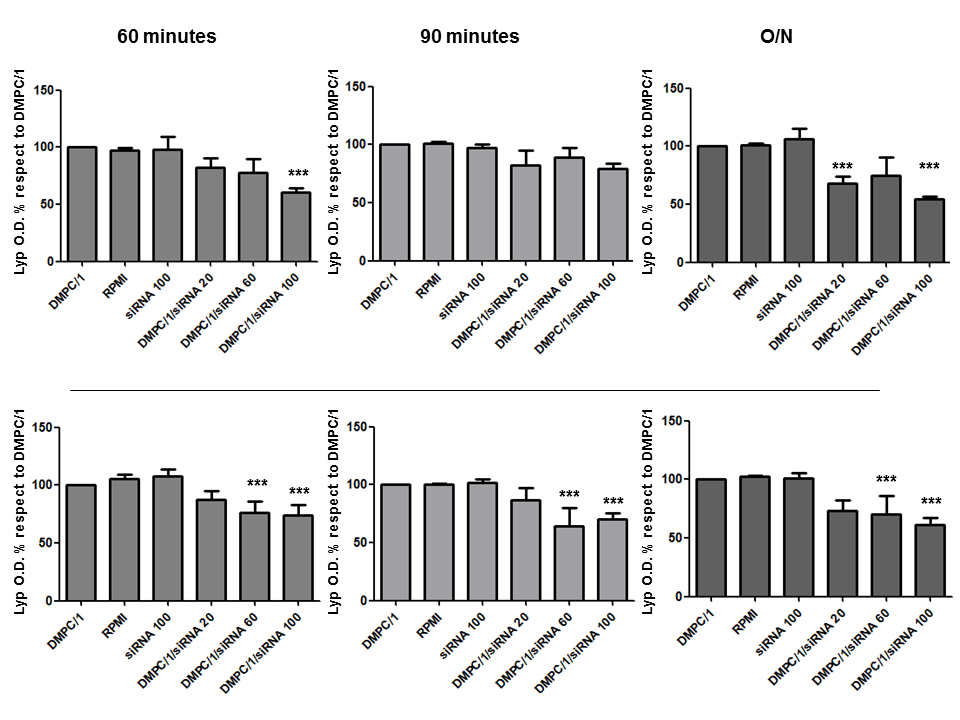

Supplement: S4 Fig — Graphs show the analysis of Lyp protein expression 48 (upper panel) and 72 hours (lower panel) after the three different transfection periods in all control and lipoplexes treated groups. *** indicates a significant statistical difference (p<0.05) between lipoplexes treated Jurkat T cells and the control groups (DMPC/1; RPMI; siRNA100). DMPC/1: n = 9; RPMI: n = 7; siRNA100: n = 5; DMPC/1/siRNA20: n = 5; DMPC/1/siRNA60: n = 5; DMPC/1/siRNA100: n = 5. (TIF) [file pone.0175784.s004.TIF]

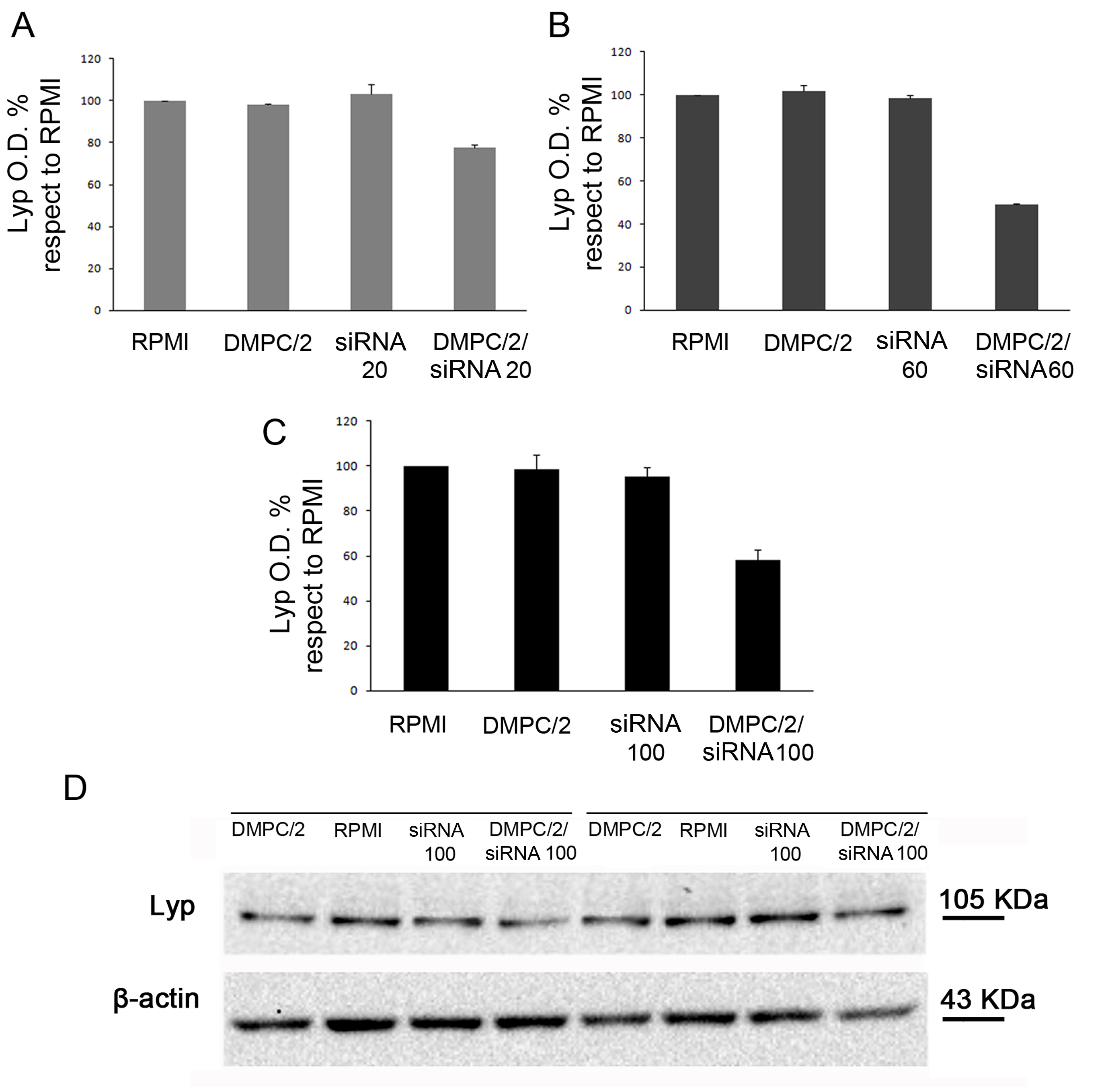

Supplement: S5 Fig — (A) Lyp expression in Jurkat T cells cultured in RPMI or after O/N transfection with DMPC/2, 20 pmols of siRNA (siRNA20) and 20 pmols of siRNA complexed in DMPC/2/siRNA lipoplexes (DMPC/2/siRNA20). 20 pmols of siRNA in DMPC/2/siRNA lipoplexes resulted in a 23% reduction of Lyp expression. (B) Same experiment as in A using 60 pmols of siRNA in DMPC/2/siRNA lipoplexes (DMPC/2/siRNA60). A 51% reduction of Lyp expression was obtained. (C) Same experiment as in A using 100 pmols of siRNA s/a in DMPC/2/siRNA (DMPC/2/siRNA100). A 42% reduction of Lyp expression was obtained. (D) Representative WB image with all experimental groups in two biological replicas is shown. Lyp O.D. values for every treatment were normalized with the corresponding β-actin values. All percentages were expressed relatively to untransfected cells (RPMI) that is considered the 100% of basal Lyp expression. Graphs A, B, C show the mean values and their standard deviations. (TIF) [file pone.0175784.s005.tif]

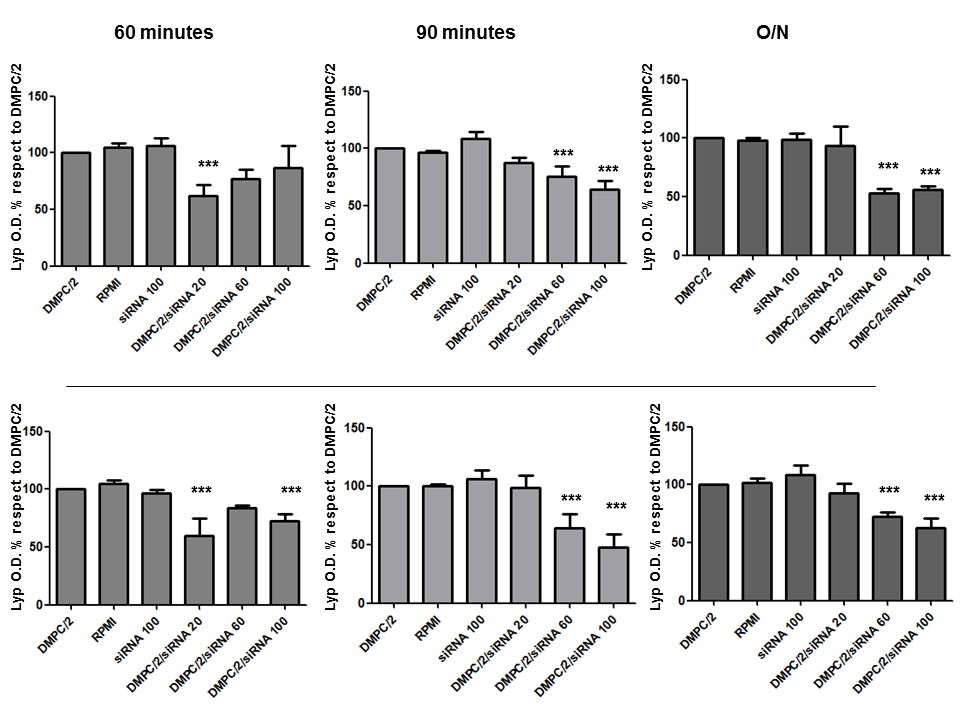

Supplement: S6 Fig — Lyp protein expression 48 (upper panel) and 72 hours (lower panel) after transfection periods in all control and lipoplexes treated groups. DMPC/2: n = 8; RPMI: n = 7; siRNA100: n = 4; DMPC/2/siRNA20: n = 4; DMPC/2/siRNA60: n = 4; DMPC/2/siRNA100: n = 4. (TIF) [file pone.0175784.s006.TIF]

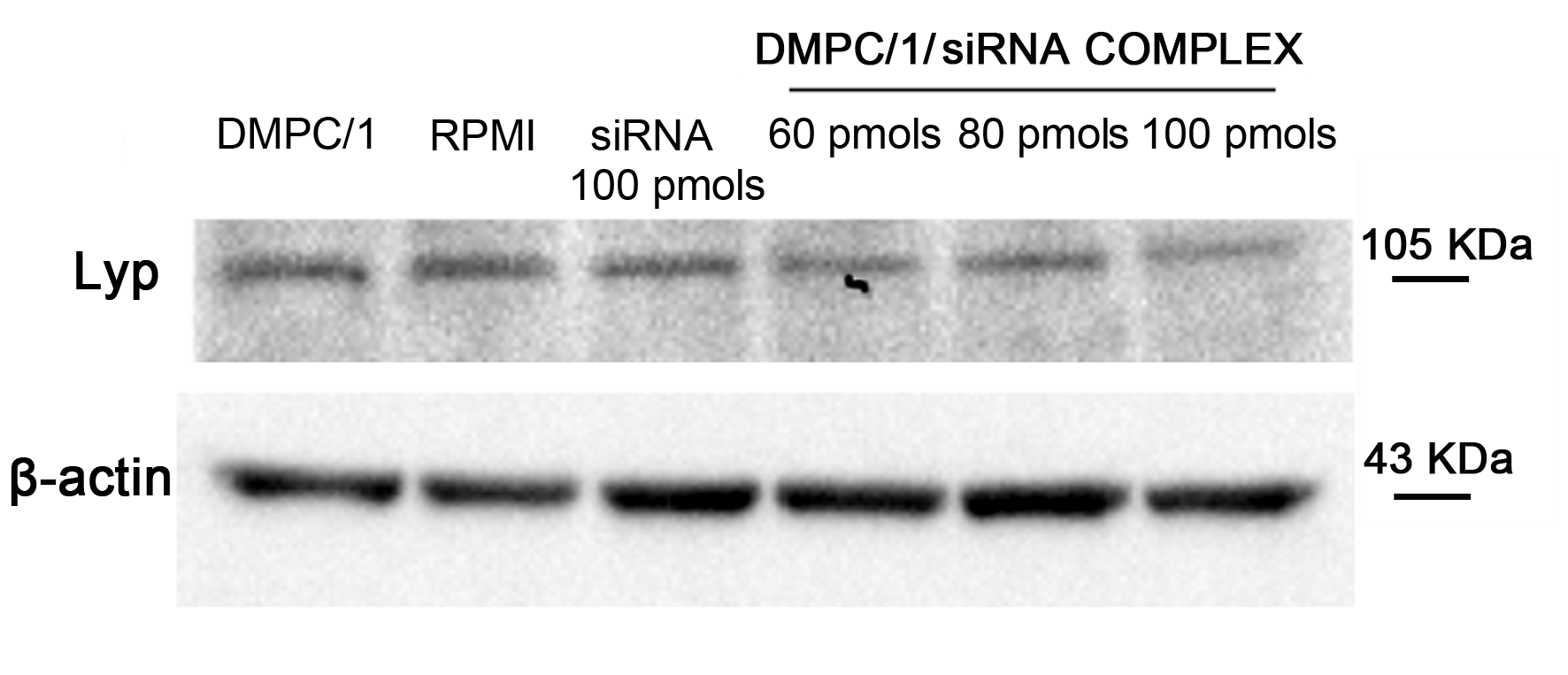

Supplement: S7 Fig — Figure shows representative WB image with all experimental groups. Cells were transfected with empty liposome, 100 pmols of siRNA alone, DMPC/1/siRNA 60, 80 and 100 pmols or cultured in RPMI. (TIF) [file pone.0175784.s007.tif]

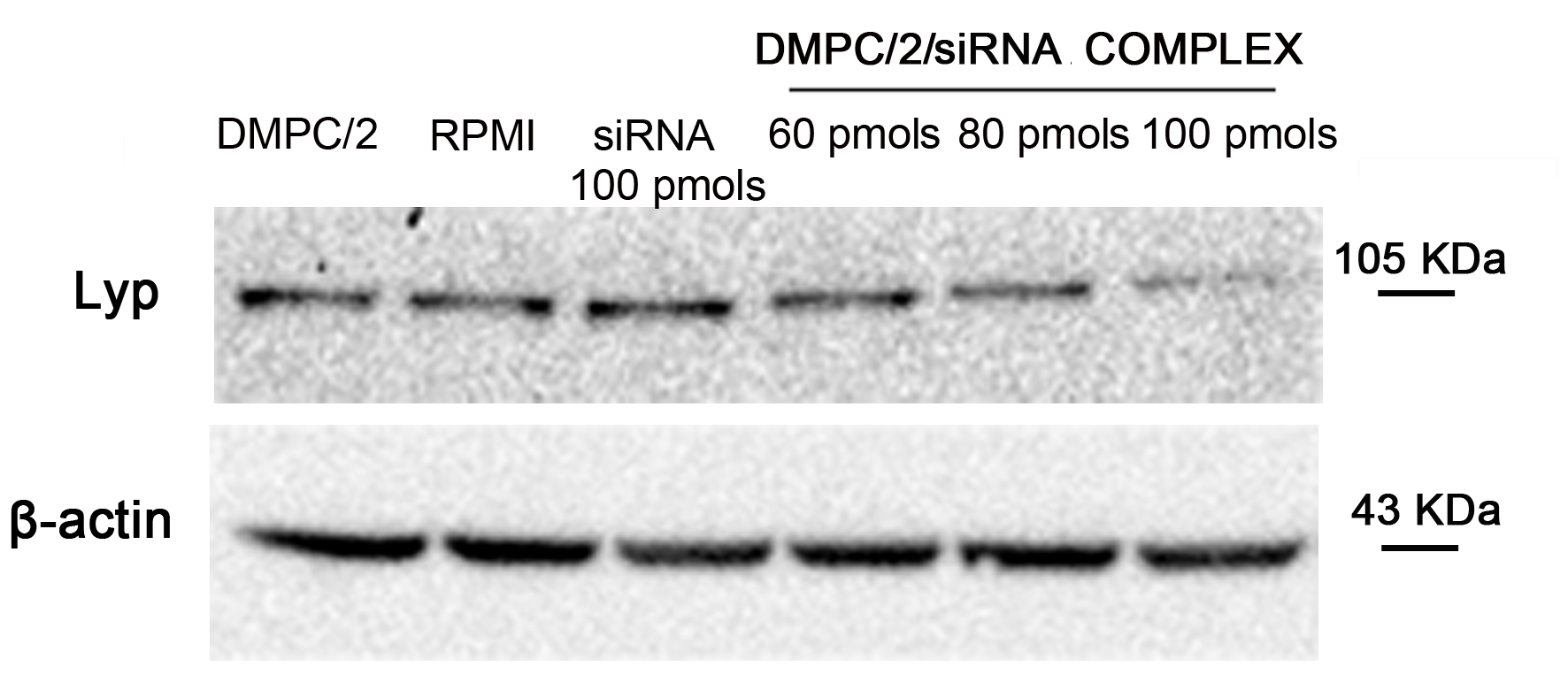

Supplement: S8 Fig — (TIF) [file pone.0175784.s008.tif]

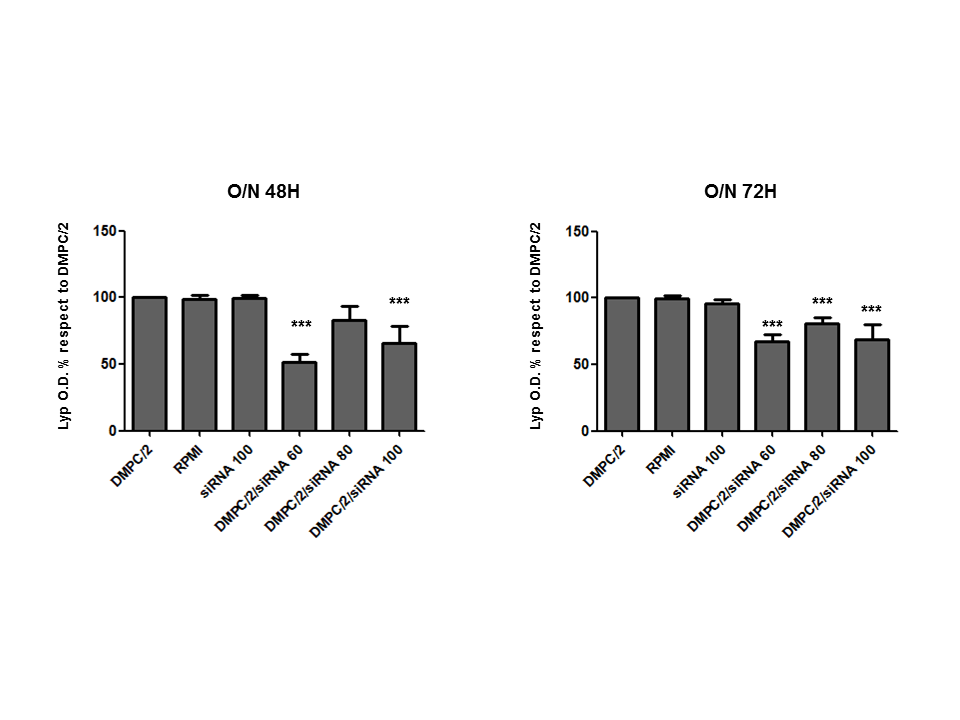

Supplement: S9 Fig — Lyp protein expression 48 and 72 hours after the O/N transfection period in all control and lipoplexes treated groups. DMPC/2: n = 7; RPMI: n = 7; siRNA100: n = 3; DMPC/2/siRNA60: n = 3; DMPC/2/siRNA80: n = 3; DMPC/2/siRNA100: n = 3. (TIF) [file pone.0175784.s009.TIF]

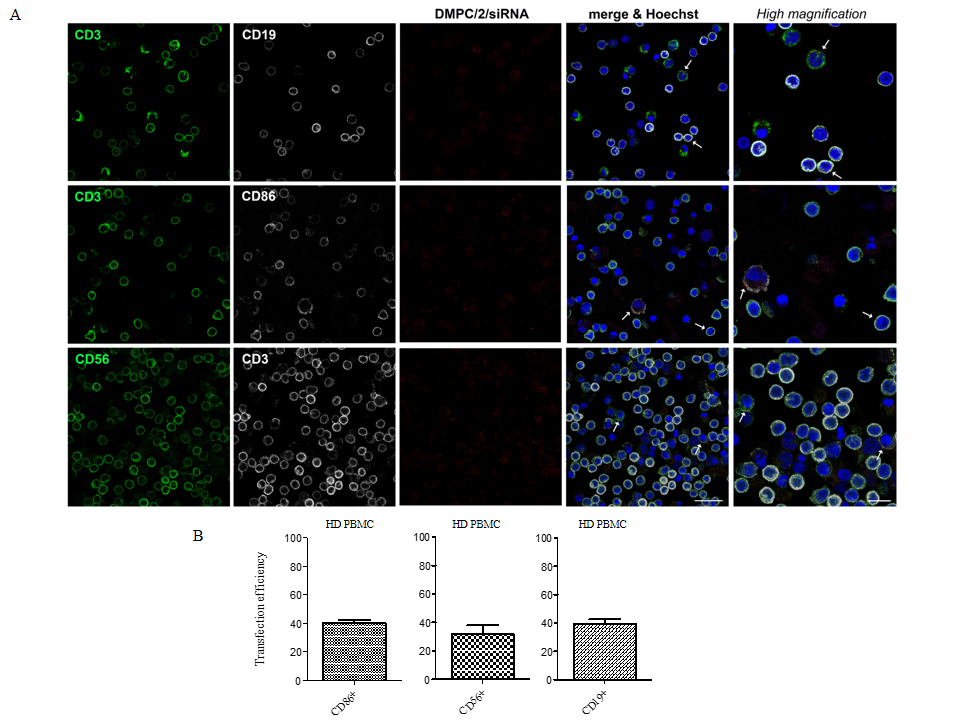

Supplement: S11 Fig — HD PBMC were administered with the indicated formulation marked with rhodamine and relative controls. After 4 and a half hours of treatment cells were fixed and stained for confocal microscopy. (A) Three stainings were performed on PBMC by using: FITC anti human CD3 (green) antibody to specifically label T cells, and Alexa Fluor 700 anti-human CD19 (white) to mark B cells; FITC anti human CD3 (green) antibody and APC anti human CD86 (white) to detect antigen presenting cells; Cy5 anti human CD3 (white) and FITC anti human CD56 (green) to stain NK cells. Cell nuclei were counterstained with Hoechst (blue). Arrows indicate lipoplexes incorporation (red dot spots). Bar: 20 μm. High magnification was shown for an improved visualization of dot internalization. Bar: 10 mm. (B) The histograms indicate the transfection efficiency through percentage of siRNA+ cells among the indicated subsets analyzed. The separate histograms represent the mean for each population analyzed and label the standard deviation. (TIF) [file pone.0175784.s011.TIF]

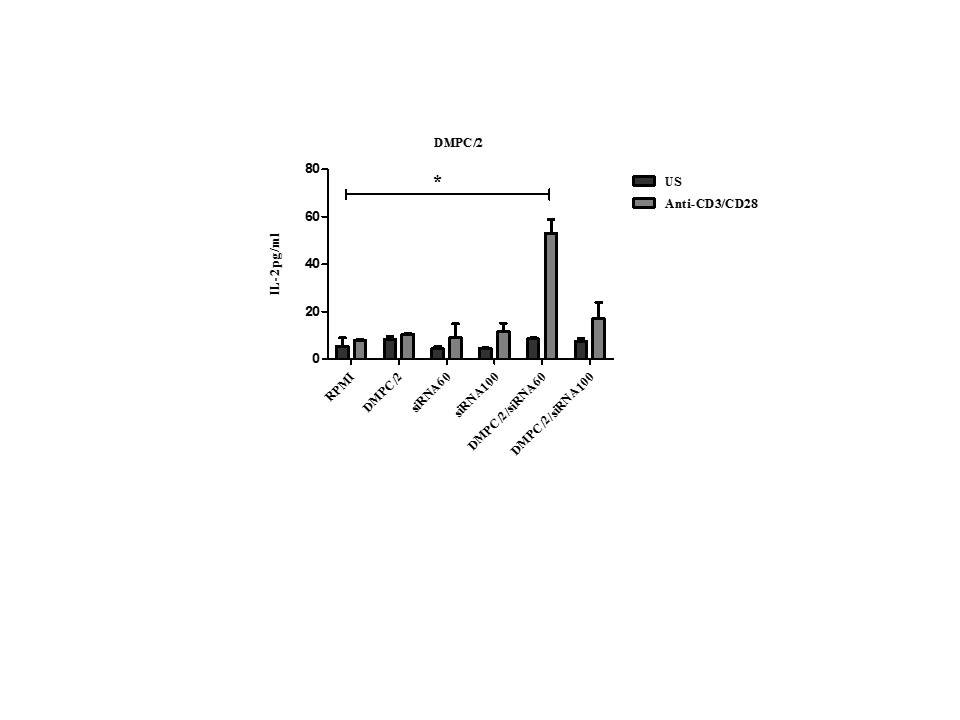

Supplement: S12 Fig — Histogram shows the increase in IL-2 production from anti-CD3/CD28 beads treated Jurkat cells specifically after DMPC/2/siRNA administration at different doses. * indicates p<0.05. (TIF) [file pone.0175784.s012.TIF]

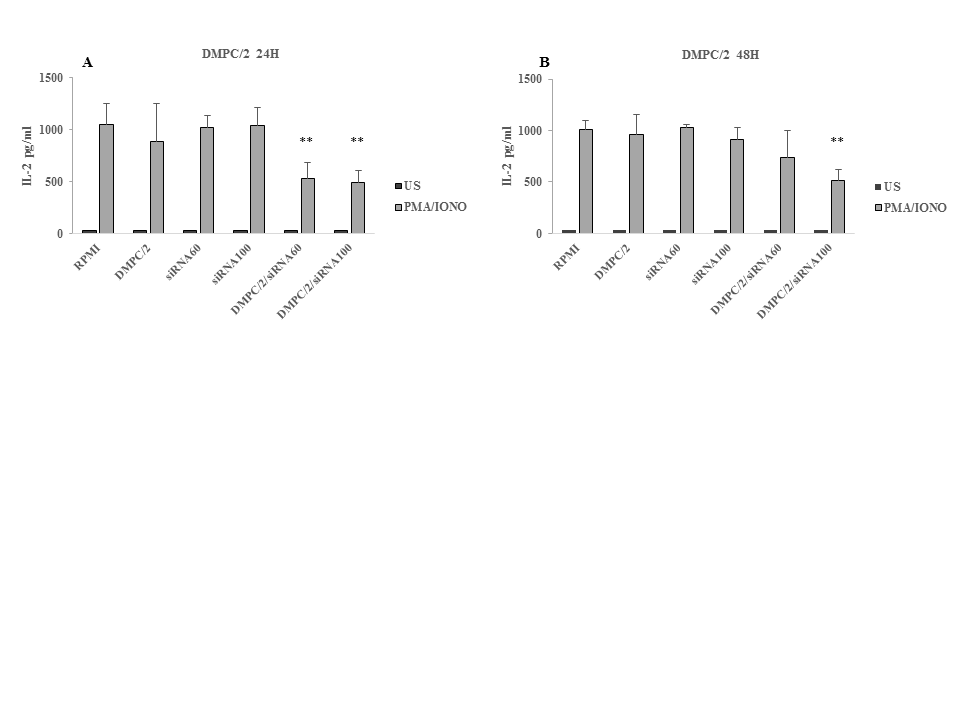

Supplement: S13 Fig — Histograms illustrate the unresponsiveness of IL-2 upon 24 (A, C) or 48 (B, D) hours of PMA/IONO (PMA-Ionomycin) stimulation in Jurkat T cells previously transfected with DMPC/2 liposomal formulation. US = control unstimulated cells; H = hours; PMA/IONO = transfected Jurkat T cells following activation with PMA/IONO. ** indicates p<0.01 (TIF) [file pone.0175784.s013.TIF]
