# Supplementary material for: Use of short interfering RNA delivered by cationic liposomes to enable efficient down-regulation of PTPN22 gene in human T lymphocytes
Source: PLoS One. 2017 Apr 24;12(4):e0175784. doi: 10.1371/journal.pone.0175784 (PMC5402975; doi:10.1371/journal.pone.0175784)
Supplement: S1 Table — Table reports the design of duplex siRNAs SNP_C (s and a) against the wild type PTPN22 allele. SNPs were designed by genetists at Sigma Chemical Co. (DOCX) [file pone.0175784.s014.docx]

|  | **Target Name** | **Sense/Antisense** | **siRNA Design** | **Start** | **Target Sequence** |
| --- | --- | --- | --- | --- | --- |
| **siRNA1** | SNP_C | s | GUACGGACACCUGAAUCAUdTdT | 45 | GTA**C**GGACACCTGAATCAT |
|  | SNP_C | a | AUGAUUCAGGUGUCCGUACdTdT | 45 | ATGATTCAGGTGTCCGTAC |
| **siRNA2** | SNP_C | s | CGGACACCUGAAUCAUUUAdTdT | 48 | **C**GGACACCTGAATCATTTA |
|  | SNP_C | a | UAAAUGAUUCAGGUGUCCGdTdT | 48 | TAAATGATTCAGGTGTCCG |
|  | SNP_C | s | ACGGACACCUGAAUCAUUUdTdT | 47 | A**C**GGACACCTGAATCATTT |
|  | SNP_C | a | AAAUGAUUCAGGUGUCCGUdTdT | 47 | AAATGATTCAGGTGTCCGT |
|  | SNP_C | s | UCCACUUCCUGUACGGACAdTdT | 35 | TCCACTTCCTGTA**C**GGACA |
|  | SNP_C | a | UGUCCGUACAGGAAGUGGAdTdT | 35 | TGTCCGTACAGGAAGTGGA |
|  | SNP_C | s | UACGGACACCUGAAUCAUUdTdT | 46 | TA**C**GGACACCTGAATCATT |
|  | SNP_C | a | AAUGAUUCAGGUGUCCGUAdTdT | 46 | AATGATTCAGGTGTCCGTA |
|  | SNP_C | s | CUUCCUGUACGGACACCUGdTdT | 39 | CTTCCTGTA**C**GGACACCTG |
|  | SNP_C | a | CAGGUGUCCGUACAGGAAGdTdT | 39 | CAGGTGTCCGTACAGGAAG |
