# Supplementary material for: Use of short interfering RNA delivered by cationic liposomes to enable efficient down-regulation of PTPN22 gene in human T lymphocytes
Source: PLoS One. 2017 Apr 24;12(4):e0175784. doi: 10.1371/journal.pone.0175784 (PMC5402975; doi:10.1371/journal.pone.0175784)
Supplement: S2 Table — Results obtained after 48 (A, C) and 72 hours (B, D) from the beginning of the transfection are listed. (DOCX) [file pone.0175784.s015.docx]

**A**

| **DMPC/1** | Percentages of Lyp expression | | |
| --- | --- | --- | --- |
|  |  |  |  |
| Treatment | O/N | | |
|  |  | | |
| RPMI | 100 | | |
| DMPC/1 | 98.5 | | |
| siRNA 100 pmols | 99.4 | | |
| DMPC/1/siRNA 100 pmols | 84.6 | | |
|  |  |  |  |

**B**

| **DMPC/1** | Percentages of Lyp expression | | |
| --- | --- | --- | --- |
|  |  |  |  |
| Treatment | O/N | | |
|  |  | | |
| RPMI | 100 | | |
| DMPC/1 | 104.8 | | |
| siRNA 100 pmols | 106.5 | | |
| DMPC/1/siRNA 100 pmols | 67.2 | | |
|  |  |  |  |

**C**

| **DMPC/2** | Percentages of Lyp expression | | |
| --- | --- | --- | --- |
|  |  |  |  |
| Treatment | O/N | | |
|  |  | | |
| RPMI | 100 | | |
| DMPC/2 | 103.2 | | |
| siRNA 100 pmols | 100.3 | | |
| DMPC/2/siRNA 100 pmols | 40.2 | | |
|  |  |  |  |

**D**

| **DMPC/2** | Percentages of Lyp expression | | |
| --- | --- | --- | --- |
|  |  |  |  |
| Treatment | O/N | | |
|  |  | | |
| RPMI | 100 | | |
| DMPC/2 | 104.3 | | |
| siRNA 100 pmols | 92.2 | | |
| DMPC/2/siRNA 100 pmols | 48.2 | | |
|  |  |  |  |
