# Supplementary material for: Use of short interfering RNA delivered by cationic liposomes to enable efficient down-regulation of PTPN22 gene in human T lymphocytes
Source: PLoS One. 2017 Apr 24;12(4):e0175784. doi: 10.1371/journal.pone.0175784 (PMC5402975; doi:10.1371/journal.pone.0175784)
Supplement: S3 Table — Values of median fluorescence intensity (MFI) indicative of proliferation obtained from CMFDA-labelled Jurkat T cells after 48 and 72 hours from the beginning of transfection with DMPC/2 lipoplexes respect to untransfected cells. (DOCX) [file pone.0175784.s016.docx]

| Treatment | MFI 48 hours | MFI 72 hours |
| --- | --- | --- |
| RPMI | 2.135 | 1.294 |
| DMPC/2 | 2.192 | 1.294 |
| siRNA 100 pmols | 2.382 | 1.410 |
| DMPC/2/siRNA100 pmols | 2.330 | 1.343 |
